# Sex differences in the effects of high fat diet on underlying neuropathology in a mouse model of VCID

**DOI:** 10.1186/s13293-023-00513-y

**Published:** 2023-05-19

**Authors:** Charly Abi-Ghanem, Abigail E. Salinero, David Kordit, Febronia M. Mansour, Richard D. Kelly, Harini Venkataganesh, Nyi-Rein Kyaw, Olivia J. Gannon, David Riccio, Gabrielle Fredman, Yannick Poitelon, Sophie Belin, Ashley M. Kopec, Lisa S. Robison, Kristen L. Zuloaga

**Affiliations:** 1 Department of Neuroscience & Experimental Therapeutics, Albany Medical College, 47 New Scotland Avenue, MC-136, Albany, NY 12208 USA; 2 Department Molecular and Cellular Physiology, Albany Medical College, 47 New Scotland Avenue, MC-136, Albany, NY 12208 USA; 3 Department of Psychology & Neuroscience, Nova Southeastern University, 3301 College Avenue, Fort Lauderdale, FL 33314 USA

**Keywords:** Diet-induced obesity, High fat diet, Prediabetes, Sex, Vascular contributions to cognitive impairment and dementia, White matter, Neuroinflammation

## Abstract

**Background:**

Damage to the cerebral vasculature can lead to vascular contributions to cognitive impairment and dementia (VCID). A reduction in blood flow to the brain leads to neuropathology, including neuroinflammation and white matter lesions that are a hallmark of VCID. Mid-life metabolic disease (obesity, prediabetes, or diabetes) is a risk factor for VCID which may be sex-dependent (female bias).

**Methods:**

We compared the effects of mid-life metabolic disease between males and females in a chronic cerebral hypoperfusion mouse model of VCID. C57BL/6J mice were fed a control or high fat (HF) diet starting at ~ 8.5 months of age. Three months after diet initiation, sham or unilateral carotid artery occlusion surgery (VCID model) was performed. Three months later, mice underwent behavior testing and brains were collected to assess pathology.

**Results:**

We have previously shown that in this VCID model, HF diet causes greater metabolic impairment and a wider array of cognitive deficits in females compared to males. Here, we report on sex differences in the underlying neuropathology, specifically white matter changes and neuroinflammation in several areas of the brain. White matter was negatively impacted by VCID in males and HF diet in females, with greater metabolic impairment correlating with less myelin markers in females only. High fat diet led to an increase in microglia activation in males but not in females. Further, HF diet led to a decrease in proinflammatory cytokines and pro-resolving mediator mRNA expression in females but not males.

**Conclusions:**

The current study adds to our understanding of sex differences in underlying neuropathology of VCID in the presence of a common risk factor (obesity/prediabetes). This information is crucial for the development of effective, sex-specific therapeutic interventions for VCID.

**Supplementary Information:**

The online version contains supplementary material available at 10.1186/s13293-023-00513-y.

## Abstract (plain language summary)

Reduced blood flow to the brain resulting from damaged blood vessels can lead to vascular dementia. Neuroinflammation and white matter damage are characteristics of vascular dementia. Middle-age is a time when obesity and prediabetes can increase risk for vascular dementia. This increase in risk is greater for women. A high fat diet causes obesity and prediabetes in mice. We compared the effects of diet-induced obesity in middle-age between males and females in a mouse model of vascular dementia. We have previously shown that a high fat diet causes greater obesity and prediabetes and a wider array of learning and memory problems in females compared to males. Here, we report on sex differences in the damage to the brain. White matter was negatively impacted by vascular dementia in males and high fat diet in females, with more severe prediabetes correlating with less white matter markers in females only. High fat diet led to an increase in activation of microglia (immune cells in the brain) in males but not in females. High fat diet also led to a decrease in pro-inflammatory and pro-resolving mediators expression in females but not males. The current study adds to our understanding of sex differences in underlying damage to the brain caused by vascular dementia in the presence of common risk factors (obesity and prediabetes). This information is needed for the development of effective, sex-specific treatments for vascular dementia.

**Supplementary Information:**

The online version contains supplementary material available at 10.1186/s13293-023-00513-y.

## Highlights

In a mouse model of comorbid VCID and metabolic disease:

- White matter was negatively impacted by cerebral hypoperfusion in males and by high fat diet in females.
- High fat diet led to an increase in activation of microglial cells (Iba1+CD68+ cells) in males but not in females.
- In both sexes, an increase in activated microglia was associated with worse episodic-like memory.
- High fat diet led to a decrease in mRNA expression of proinflammatory cytokines and pro-resolving factors in females but not in males.

**Supplementary Information:**

The online version contains supplementary material available at 10.1186/s13293-023-00513-y.

## Background

Vascular contributions to cognitive impairment and dementia (VCID) is the second most common cause of dementia [1]. Metabolic diseases, such as obesity and diabetes, are well-known risk factors for VCID. Obesity, especially at mid-life, has been shown to increase VCID risk by up to fivefold independent of cardiovascular disease, stroke and diabetic status [2]. Moreover, obesity has been associated an increased severity of vascular risk factors that worsen VCID pathology [5]. Type 2 diabetes is also a well-known risk factor for VCID, as it causes damage to the cerebral vasculature and blood brain barrier [3–5]. Prediabetes, defined as impaired glucose tolerance and/or slightly increased fasting glucose, has similar negative effects on cerebrovascular disease, such as promoting endothelial dysfunction and arterial stiffness [6]. Prediabetes is even more common than type 2 diabetes. It is estimated that 34% of the US population is prediabetic, though prediabetes often goes undiagnosed [7]. Prediabetes has been shown to increase the risk of VCID by ~ 50% [8]. Damage to cerebral vessels, whether arising from prediabetes, obesity, or other pathological contributors to VCID, creates a state of cerebral hypoxia accompanied by neuroinflammation [9–11]. This leads to disruption and degradation of the white matter and even death of oligodendrocytes (the myelin forming cells of the CNS) [9, 12]. Both prediabetes and obesity have been associated with white matter damage [13–21] and neuroinflammation [11, 22–25].

We have previously shown that obesity and prediabetes, modeled using chronic administration of a high fat (HF) diet, causes cognitive impairment in the context of normal aging [26] and in a mouse model of VCID [27]. However, these were single-sex studies. VCID is more common in men than women throughout most of the lifespan; however, this sex difference is reversed in the presence of certain comorbidities, such as metabolic disease. In fact, diabetic women have a 19% greater risk of developing VCID than diabetic men [28]. Prediabetes is also associated with executive function decline in women but not men [29]. Given the increasing prevalence of prediabetes and obesity as well as the known sex difference in the risk of developing cognitive impairment/VCID within the (pre)/diabetic population, it is important to understand how sex may influence the degree to which obesity and prediabetes impact VCID.

The goal of the current study was to determine if there are sex differences in the effects of mid-life metabolic disease in mouse model of VCID. We used chronic administration of a HF diet to induce obesity and prediabetes, and a unilateral common carotid artery occlusion surgery to induce chronic cerebral hypoperfusion and model VCID. We previously reported that these middle-aged HF-fed females had greater metabolic impairment (weight gain, glucose intolerance, visceral fat accumulation), and a wider array of cognitive deficits (novel object recognition, Morris water maze) compared to HF-fed males (novel object recognition impairment only) [30]. VCID also caused a wider array of cognitive deficits in females compared to males [30]. Here, we investigated sex differences in the underlying neuropathology focusing on two hallmarks of VCID: white matter changes and neuroinflammation. We identified sex differences in both types of neuropathology, further highlighting the need to consider sex as a key biological variable.

## Materials and methods

### Animals and experimental design

**Fig. 1:**
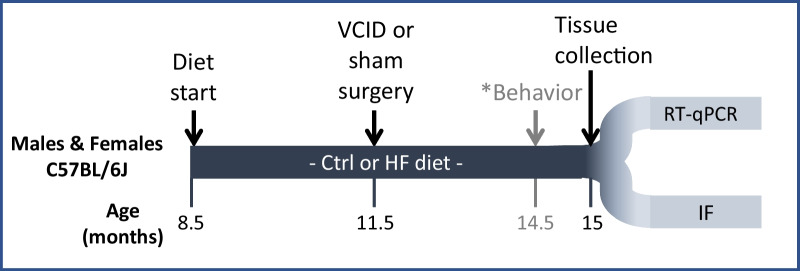
Experimental design. Experimental timeline. *Ctrl* control diet (10% fat), *HF* high fat diet (60% fat), *VCID* vascular contributions to cognitive impairment and dementia, *RT-qPCR* real time quantitative polymerase chain reaction, *IF* immunofluorescent labeling. *Results of the behavioral testing can be found in Salinero et al. [30]

All experiments were approved by the Albany Medical College Animal Care and Use Committee and in compliance with the ARRIVE guidelines. Male and female C57BL/6J mice (~ 8.5 months old) were obtained from Jackson Laboratories (Bar Harbor, ME, USA). Mice were housed (3–5 per cage) at 21 °C, 30–70% humidity, with a 12 h light/dark cycle. After 1 week of acclimation and for the remainder of the study, cages of mice were randomized to treatment groups and placed on either a high fat (HF) diet (60% fat, D12492, Research Diets, New Brunswick, NJ, USA) or a control (Ctrl) diet (10% fat, D12450B, Research Diets, USA). Three months after diet onset, mice underwent a right unilateral common carotid artery occlusion (UCCAO) to model VCID or a sham surgery, as previously described [30]. Three months after surgery, cognitive deficits were tested. Details of procedures, as well as metabolic and behavioral results, were previously published [30]. At the end of the study, mice (~ 15 months old) were deeply anesthetized with pentobarbital and intracardially perfused with saline. Brains were collected and further processed according to downstream procedure. A total of 160 mice (20/group) were used in the study. Six mice died during surgery, and an additional 14 were excluded as they died prematurely or had to be euthanized due to illness. The remaining 140 brains were randomly distributed among 3 analysis groups, 2 of which are presented in this study. Brains were either post-fixed for IHC or regionally dissected and flash frozen for RT-qPCR. Further, animals were excluded for biological reasons such as tumors or evidence of stroke (5 animals; 2 male HF VCID, 3 female HF VCID); and for technical reasons such as poor RNA yield/quality or tears/folds in the tissue for IHC. A timeline of the project is shown in Fig. 1.

### Immunohistochemistry

Brains were collected and fixed overnight in a 4% paraformaldehyde (PFA) solution and then cryoprotected in 30% sucrose then frozen in optimal cutting temperature (O.C.T.) solution (23-730-571, Thermo Fisher Scientific) and stored at − 80 °C until further processing. Brains were sectioned in the coronal plane into six series of 40-μm-thick sections using a cryostat (Cm1950, Leica). One series of brain slices was immunolabeled for myelin and oligodendrocytes using the following protocol. On day 1, the slices were washed with PBS and then permeabilized and blocked, using PBS with 0.3% triton (TPBS) and 5% donkey serum solution, for one hour at room temperature. Next, a preincubated mixture of the following antibodies was added: primary mouse anti-CC1 (1:500, Millipore OP80) and secondary donkey anti-mouse CY5 (1:10, 715-605-150 Jackson ImmunoResearch) in normal mouse serum (015-000-001 Jackson ImmunoResearch) and incubated in a cold room overnight. The CC1 antibody, originally developed for adenomatous polyposis coli, recognizes Quaking 7 an RNA-binding protein that is highly up-regulated in myelinating oligodendrocytes in the central nervous system [31]. On day 2, the slices were washed with PBS with 0.01% sodium azide and then incubated with a chicken anti-MBP primary antibody (1:1000; A1-10,008 Invitrogen) overnight in a cold room. On day 3, the slices were washed with PBS and then a mixture of donkey anti-chicken 488 (1:1000; 703-545-155 Jackson ImmunoResearch) and DAPI (1:1000) were added, incubated for 1 h, and then washed with PBS with sodium azide and mounted using ProLong Gold Antifade Mountant (P36930 Thermo Fisher Scientific). Another series of brain slices was immunolabeled to assess microglia activity as previously described [19–21]. Briefly, slices were washed with PBS and then permeabilized and blocked, using a 0.3% PBS with triton (TPBS) and 5% donkey serum solution, for one hour at room temperature. Primary antibodies of goat anti-Iba1 (1:1000; PA5-18039, lot #TI2638761, Thermo Fisher Scientific) and rat anti-CD68 (1:1000; MCA1957, lot #1708, Bio-Rad) were applied overnight at 4 °C. Rhodamine Red-X donkey anti-rat (1:100; 712-295-150 Jackson ImmunoResearch) and Alexa Fluor 647 donkey anti-goat (1:300; 705-605-147, Jackson ImmunoResearch) were added in blocking buffer for 2 h at room temperature.

### Image analysis

Images of brain slices were obtained at 10× magnification using the Axio Observer Fluorescent Microscope (Carl Zeiss Microscopy, Oberkochen, Germany). All analyses were conducted using coronal brain slices with the posterior corpus callosum, the fornix, and the hippocampus visible between bregma − 1.28 to − 1.82. Images were imported to NIH’s ImageJ software. Regions of interest (ROIs) were drawn around the corpus callosum, and the entire hippocampus or the CA1 region. On average, a minimum of three brain slices containing each ROI was used and measurements were averaged across the brain slices for each animal. All measurements were made by an experimenter who was blinded to treatment group.

#### Quantification of myelin and oligodendrocytes

For myelin and mature oligodendrocyte analysis, respectively, MBP and CC1 positive area density was obtained for each ROI and average density (average of three brain slices per ROI per animal) were compared in the right hemispheres (the side of the UCCAO) between animals across the four experimental groups. For the MBP analysis of the medial corpus callosum, a rectangular ROI was used for optimal thresholding. All analyses were performed in the right hemisphere of coronal sections by an experimenter who was blinded to treatment group using ImageJ (NIH). Representative images are shown in Additional file 1: Fig. S1.

#### Quantification of microglia-related measures

Iba1 and CD68 images were thresholded using ZEN (blue edition, Carl Zeiss Microscopy) software. Regions of interest (ROIs) were drawn around the medial corpus callosum and the cornu ammonis 1 (CA1) region of the hippocampus. Cells were counted manually, and microglia (Iba1+ cells) were classified into 4 cell types according to morphology and immunoreactivity to CD68: ramified (punctate cell body) with and without CD68, and ameboid (larger cell body; few or no processes) with and without CD68. Additionally, we have quantified the area density occupied by the Iba1 labeling in the CC and the entire hippocampus. All analyses were performed in the right hemisphere of coronal sections by an experimenter who was blinded to treatment group using ImageJ (NIH). Representative images could be seen in Additional file 1: Fig. S3.

### RT-qPCR

The hippocampus was extracted in ice cold PBS, flash frozen and stored at − 80 °C. The hippocampus was homogenized in 50 µL of RNA later (R0901 Sigma) and half of the homogenate was used to extract RNA using Qiagen RNeasy plus mini kit according to the manufacturer’s instructions (74131 Qiagen). RNA concentration was determined using a nanodrop and 1ug of this was converted to cDNA using High-Capacity cDNA Reverse Transcription Kit with RNase Inhibitor (4374967 Thermo Fisher Scientific) according to the manufacturer’s instructions. Quantitative PCR was performed in triplicates on 50 ng of cDNA in a 10ul reaction using TaqMan probe technology on a Bio-Rad CFX-384 real time system. Taqman assays (Thermo Fisher scientific) used were: Il1b, Mm00434228_m1; Tnfα, Mm00443258_m1; Anxa1, Mm00440225_m1; Fpr2, Mm00484464_s1; Iba1(Aif1), Mm00479862_g1; Arg1, Mm00475988_m1; CD31, Mm01242576_m1; PDGFRβ, Mm00435553_m1; Claudin5, Mm00727012_s1; Chil3 (YM1), Mm00657889_Mh; Retnla (KIZZ1), Mm00445109_m1. Rps17, Mm01314921_g1; and Rpl13a, Mm05910660_g1 were used as housekeeping genes. Bio-Rad CFX Maestro 1.1 software was used to analyze the data. The relative expression levels of genes of interest were calculated using the ΔΔCq method relative to either of the housekeeping genes using the males on a Ctrl diet with sham surgery as reference group.

### Statistics

Statistical analyses were completed using GraphPad Prism (GraphPad Software, San Diego, CA, USA). Data were checked for normal distribution and heteroscedasticity. In cases of non-conformity, values were transformed to their log2 or square root before performing a 2-way ANOVA (Additional file 1: Table S1) followed by Tukey’s post hoc test to analyze the effects of diet and VCID in each sex. In secondary analysis, 3-way ANOVA was used to test for sex differences. Statistical significance was set at *p* < 0.05. All graphs show individual data points, as well as mean + SEM. Correlations were run for all animals and separately for each sex using Pearson correlations and linear regression models.

## Results

To induce metabolic disease, a high fat (HF; 60% fat) or low fat control diet (Ctrl; 10% fat) was administered to middle-aged male and female C57BL/6J mice from 8.5 to 15 months of age. To model VCID via chronic cerebral hypoperfusion, mice (11.5 months of age) underwent a unilateral common carotid artery occlusion surgery or control (sham) surgery. At 14.5 months of age mice underwent a battery of behavior tests to assess cognitive deficits. At 15 months of age, tissue was harvested to investigate pathology (see timeline in Fig. 1). We have previously reported that these middle-aged HF-fed females had greater metabolic impairment (weight gain, glucose intolerance, visceral fat accumulation), and a wider array of cognitive deficits (novel object recognition, Morris water maze) compared to HF-fed males (novel object recognition impairment only) [30]. VCID also caused a wider array of cognitive impairment in females (both tests) compared to males (Morris water maze only) [30]. Here, we investigate underlying neuropathology associated with both metabolic disease and VCID, including white matter changes and neuroinflammation.

### White matter changes are more heavily driven by VCID in males and HF diet in females

VCID is characterized by accumulation of white matter damage that is detected as white matter hyperintensities using MRI [10]. We examined myelination in two brain regions: the corpus callosum (CC; the main white matter tract connecting both hemispheres) and hippocampus (a grey matter region that is critical for learning and memory) using immunolabeling for myelin basic protein (MBP) and CC1 to label mature oligodendrocytes. Representative images of each group are shown in Additional file 1: Fig. S1.

#### Corpus callosum

**Fig. 2:**
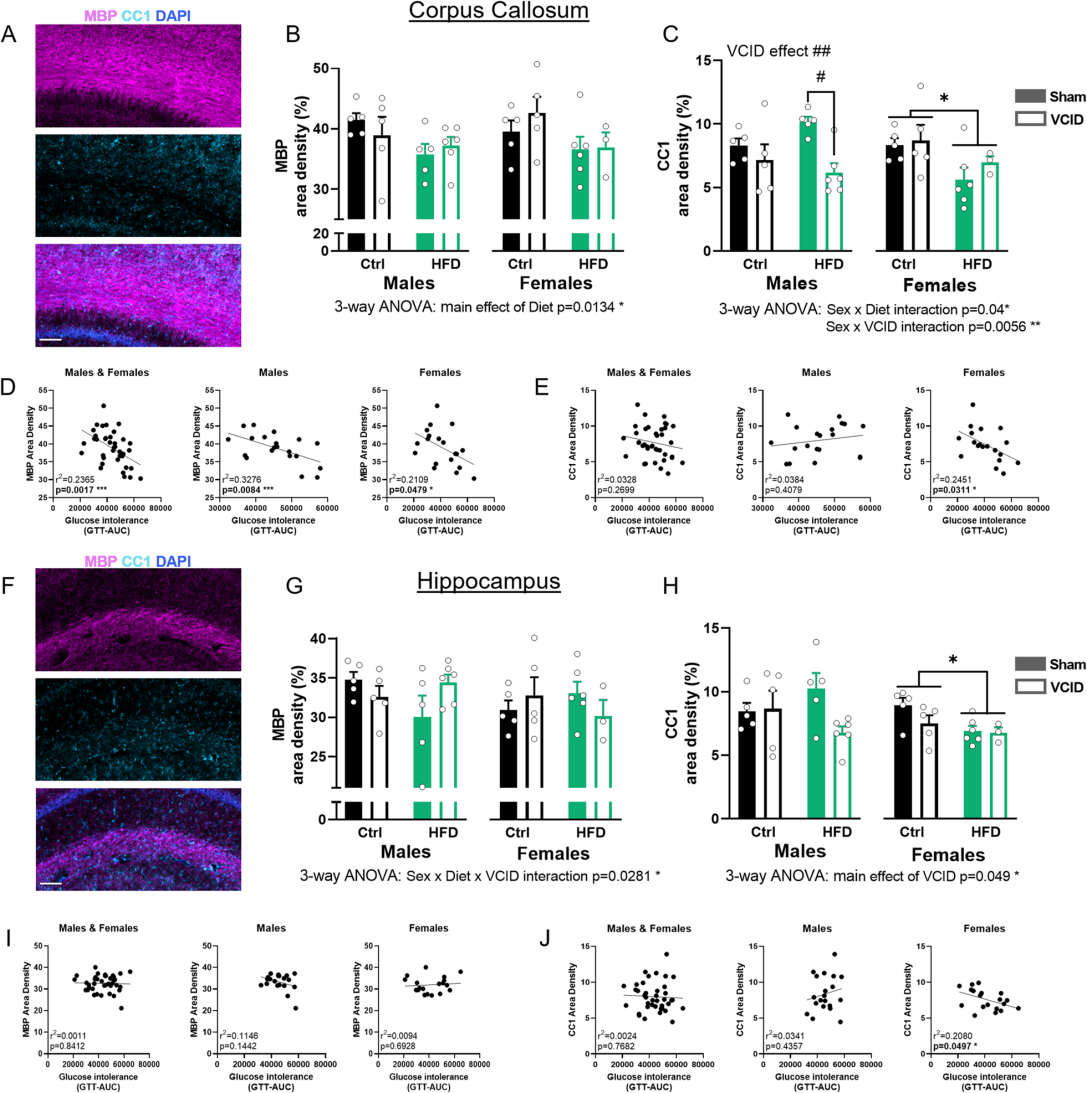
White matter changes are driven by VCID in males and HF diet in females. Representative images of myelin basic protein (MBP) labeling (top panel) and CC1 labeling (mid panel) are shown in **A** for the corpus callosum (CC) and **F** for the hippocampus. In the merged image shown in the bottom panel, MBP is shown in magenta, CC1 in cyan and DAPI in blue. Scale bar 100 µm. Labeling density quantification in the CC (**B**, **C**) and the hippocampus (**G**, **H**). Data are represented as mean + SEM. 2-way ANOVA with Tukey’s post hoc test ^#^*p* < 0.05, ^##^*p* < 0.01 effect of VCID; *p < 0.05, **p < 0.01 effect of diet. 3-way ANOVA results are reported under the graph *p < 0.05. Correlations of glucose intolerance (GTT-AUC) with MBP (**D**, **I**) and CC1 (**E**, **J**) densities were analyzed using simple linear regression for males and females combined and for each sex separately with *r*^2^ and *p* values noted for each. *n* = 3–6 mice/group. *Ctrl* control diet, *HFD* high fat diet

In the corpus callosum (Fig. 2A–C), we did not observe any significant effect of HF diet on MBP density in males or females (2-way ANOVA). However, 3-way ANOVA analysis revealed a main effect of HF diet in decreasing the % area covered by MBP (main effect of diet, *p* = 0.0134). This diet effect was detectable due to the increased statistical power of a 3-way ANOVA that considers effects across both sexes. For mature oligodendrocytes (CC1+ cells), there was no effect of HF diet in males; however, we observed a significant reduction in CC1+ density in VCID males (2-way ANOVA VCID effect *p* = 0.0034). This was further supported by a post hoc difference where for HF-fed males, VCID animals had lower density than sham controls (Tukey’s post hoc *p* = 0.0114 HF sham vs HF VCID). In females, we observed a main effect of diet in reducing mature oligodendrocyte density (2-way ANOVA diet effect *p* = 0.0315). No VCID effect was observed in females. When analyzing sex differences via 3-way ANOVA, we observed a sex × VCID interaction (*p* = 0.0056), in which VCID surgery reduced CC1 density in males but not females. This is further supported by post hoc tests, which showed that VCID surgery reduced CC1 density in HF males only (Sidak’s post hoc test, *p* = 0.0274). Conversely, there was a sex × diet interaction (*p* = 0.04) in which HF diet reduced CC1 density in females (both sham and VCID), but not in males. This is further supported by post hoc tests, which showed HF diet reduced CC1 density in sham females compared to sham males on a HF diet (3-way ANOVA with Sidak’s post hoc test, *p* = 0.0033).

Using metabolic health and behavior data from these mice (previously reported [30]), we ran correlation analysis between these measures and white matter measures. Interestingly, we found an association between the glucose tolerance test area under the curve (GTT-AUC) and levels of MBP area density when pooling both sexes (linear regression, *r*^2^ = 0.2365, *p* = 0.0017), wherein worse glucose intolerance was associated with lower myelin coverage (Fig. 2D). This negative correlation was seen in each sex (males: linear regression, *r*^2^ = 0.3276, *p* = 0.0084, females: linear regression, *r*^2^ = 0.2109, *p* = 0.0479). Conversely, when examining the relationship between glucose intolerance and mature oligodendrocyte density we do not observe any significant correlation when pooling sexes together (Fig. 2E). However, when data were segregated by sex, in females, but not males, there was a negative correlation between the glucose tolerance test area under the curve (GTT-AUC) and levels of CC1 area density (linear regression, *r*^2^ = 0.2451, *p* = 0.0311), wherein worse glucose intolerance was associated with lower mature oligodendrocyte density. No correlations were observed between white matter measures and behavior outcomes (Additional file 1: Fig. S2).

#### Hippocampus

In the hippocampus (Fig. 2F–H), a major contributor to spatial memory, we did not observe any significant effect of HF diet on MBP density in males or females (2-way ANOVA). We observed a sex × diet × VCID interaction for MBP (*p* = 0.0281); however, these differences were small. For oligodendrocytes, there was no effect of diet or VCID in males. In females, similar to the CC, we observed a main effect of HF diet in reducing cell density (2-way ANOVA main effect of diet *p* = 0.0434). There was a main effect of VCID surgery to reduce hippocampal CC1 (*p* = 0.049). No correlations were observed between white matter measures in the hippocampus and metabolic outcomes (Fig. 2I and Additional file 1: Fig. S2). Similar to the CC, we observed a negative association in females, but not males, between the glucose tolerance test area under the curve (GTT-AUC) and levels of CC1 area density (linear regression, *r*^2^ = 0.2080, *p* = 0.0497; Fig. 2J). Thus, in females more severe glucose intolerance was associated with a reduction in a maker for mature oligodendrocytes.

### HF diet increased microglia in males but not in females

**Fig. 3:**
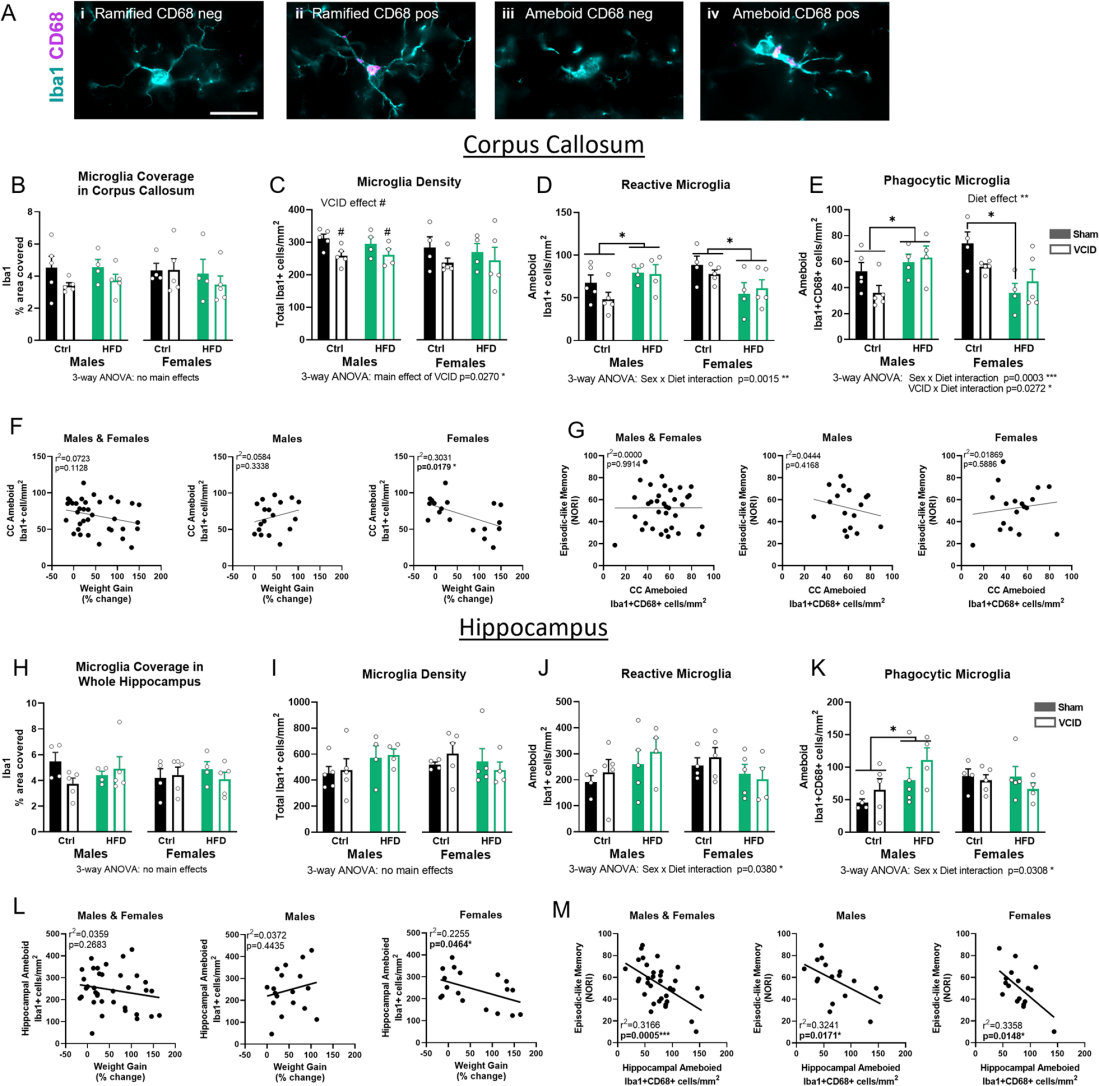
High fat diet induces microglia activation in males but suppresses it in females. Microglia were labeled using Iba1 (cyan) and CD68 (magenta). Cells were classified into 4 groups depending on morphology and CD68 immunoreactivity: ramified cells with or without CD68 and ameboid cells with or without CD68 (**A**, **i**–**iv**). Scale bar 20 µm. The % area occupied by the Iba1 labeling was quantified in the CC (**B**) and the hippocampus (**H**). Cell densities were quantified in the corpus callosum (**C**–**E**) and the CA1 region of the hippocampus (**I**–**K**). Data are represented as mean + SEM. 2-way ANOVA with Tukey’s post hoc test. ^#^*p* < 0.05 effect of VCID; *p < 0.05, **p < 0.01 effect of diet. 3-way ANOVA results are reported under the graph *p < 0.05, **p < 0.01, ***p < 0.001. Correlation of Iba1+ ameboid cell density with body weight change expressed as % of body weight at the start of the experiment in the CC (**F**) and in the CA1 of the hippocampus (**L**). Correlation of ameboid Iba1+CD68+ cell density with the novel object recognition index (NORI) as a measure of episodic-like memory in the CC (**G**) and in the CA1 of the hippocampus (**M**). Correlation analyzed using simple linear regression for each sex with *r*^2^ and *p* values noted for each. *p < 0.05, ***p < 0.001, *n* = 4–6 mice/group. *Ctrl*  control diet, *HFD* high fat diet

Microglia play a key role in the immune/inflammatory response in the CNS in several conditions, including VCID. Ionized calcium binding adapter molecule 1 (Iba1), which also labels infiltrating monocytes, is a well-accepted marker for both resting and activated microglia [32–34]. Switching from a ramified to an ameboid morphology is considered a feature of activated microglia. CD68, which labels the lysosomal membrane of microglia and macrophages, is used to identify phagocytic microglia [35, 36]. We used both markers to examine microglia number and activation state in the CC and the hippocampus. We classified microglia (Iba1+ cells) into 4 cell types according to morphology and immunoreactivity to CD68: ramified (punctate cell body) with and without CD68, and ameboid (larger cell body; few or no processes) with and without (Fig. 3A). Representative images of each group are shown in Additional file 1: Fig. S3.

#### Corpus callosum

In the CC, we did not observe any statistical differences in the area covered by the Iba1 labeling (Fig. 3B). Since microglia are known to become reactive and swell in response to stimuli, we also assessed cell number and phenotype. VCID surgery reduced microglia density in males (total Iba1+ cells/mm^2^; 2-way ANOVA main effect of VCID *p* = 0.0142) but not females (Fig. 3C). However, the 3-way ANOVA did not show a sex difference. When examining reactive microglia (Iba1+ cells with an ameboid morphology), HF diet increased cell density in the males (2-way ANOVA main effect of diet *p* = 0.035) but decreased cell density in the females (2-way ANOVA main effect of diet *p* = 0.0203). This sex difference in the effect of HF diet is supported by a sex × diet interaction (3-way ANOVA *p* = 0.0015; Fig. 3D). Microglia that are CD68 positive were characterized as “phagocytic”. In males, HF diet increased ameboid phagocytic microglia cell density (2-way ANOVA *p* = 0.0255; Fig. 3E). In females however, HF diet decreased phagocytic microglia cell density (2-way ANOVA *p* = 0.0045), which was highlighted by a significant post hoc difference between Ctrl sham females and HF sham females (Tukey’s post hoc *p* = 0.0169). This was supported by a sex × diet interaction in the number of amoeboid phagocytic microglia (3-way ANOVA *p* = 0.0003). Additionally, we observed a VCID × diet interaction (*p* = 0.0272), in which VCID mice of either sex on a control diet had decreased numbers of ameboid phagocytic microglia relative to their sham controls but VCID mice on a HF diet did not.

#### Hippocampus

Similar to the CC, we did not observe any statistical differences in the area covered by Iba1 labeling (Fig. 3H). We also counted Iba1+ cells specifically in the CA1 region of the hippocampus. We focused on the CA1 region since it has been shown to be more vulnerable to ischemia and hypoxia, which are often observed in VCID in both humans and rodent models [37–40]. Further, recent murine studies have shown that CA1 plays a key role in episodic-like memory processing through the novel object recognition task [41, 42]. When quantifying the microglial cells, no group differences were detected via 2-way ANOVA in either sex for total microglia cell density, as well as reactive microglia. However, 3-way ANOVA revealed a sex × diet interaction for reactive (ameboid) microglia cell density (*p* = 0.0380, Fig. 3J) as these cells were increased in HF-fed males but not females. Phagocytic (ameboid, CD68+Iba+) microglia were increased by HF diet in males (2-way ANOVA main effect of diet *p* = 0.0317) but not females, which is supported by a sex × diet interaction (3-way ANOVA *p* = 0.0308, Fig. 3H).

To better understand the relationship between weight gain and microglia activation, we assessed the association between weight gain and the density of reactive microglia in the CC and the hippocampus. When pooling together all mice (regardless of sex), we do not observe any association; however, when separating by sex, we observed a female-specific negative correlation in both brain regions (linear regression, CC: *r*^2^ = 0.3031, *p* = 0.0179, hippocampus: *r*^2^ = 0.2255, *p* = 0.0464). In females, greater weight gain (% change in body weight) was associated with fewer reactive microglia in the CC and hippocampus (Fig. 3F, L). We then examined whether microglia activation was associated with cognitive deficits. We did not find any significant correlations between reactive microglia and cognitive measures (Additional file 1: Fig. S4). However, when looking at the specific subpopulation of reactive microglia that are phagocytic, we observed a negative association with episodic-like memory in both sexes in the hippocampus but not CC (Fig. 3G, M). Indeed, a higher number of Iba1+CD68+ ameboid cells in the hippocampus correlated with a decrease in novel object recognition index (NORI; measure of impaired episodic-like memory; males and females combined *r*^2^ = 0.3166, *p* = 0.0005; males *r*^2^ = 0.3241, *p* = 0.0171; females *r*^2^ = 0.3358, *p* = 0.0148).

Taken together, these data show a region-specific effect of VCID on microglial cell density (decreased in CC but not hippocampus) and a sex-specific effect of the HF diet on microglia activation (increased in males, but not in females). In both sexes, regardless of diet, a greater density of ameboid phagocytic microglia in the hippocampus was associated with worse episodic-like memory.

### Hippocampal inflammatory genes were increased in males and decreased in females fed a HF diet

#### Iba1 and pro-inflammatory cytokines

**Fig. 4:**
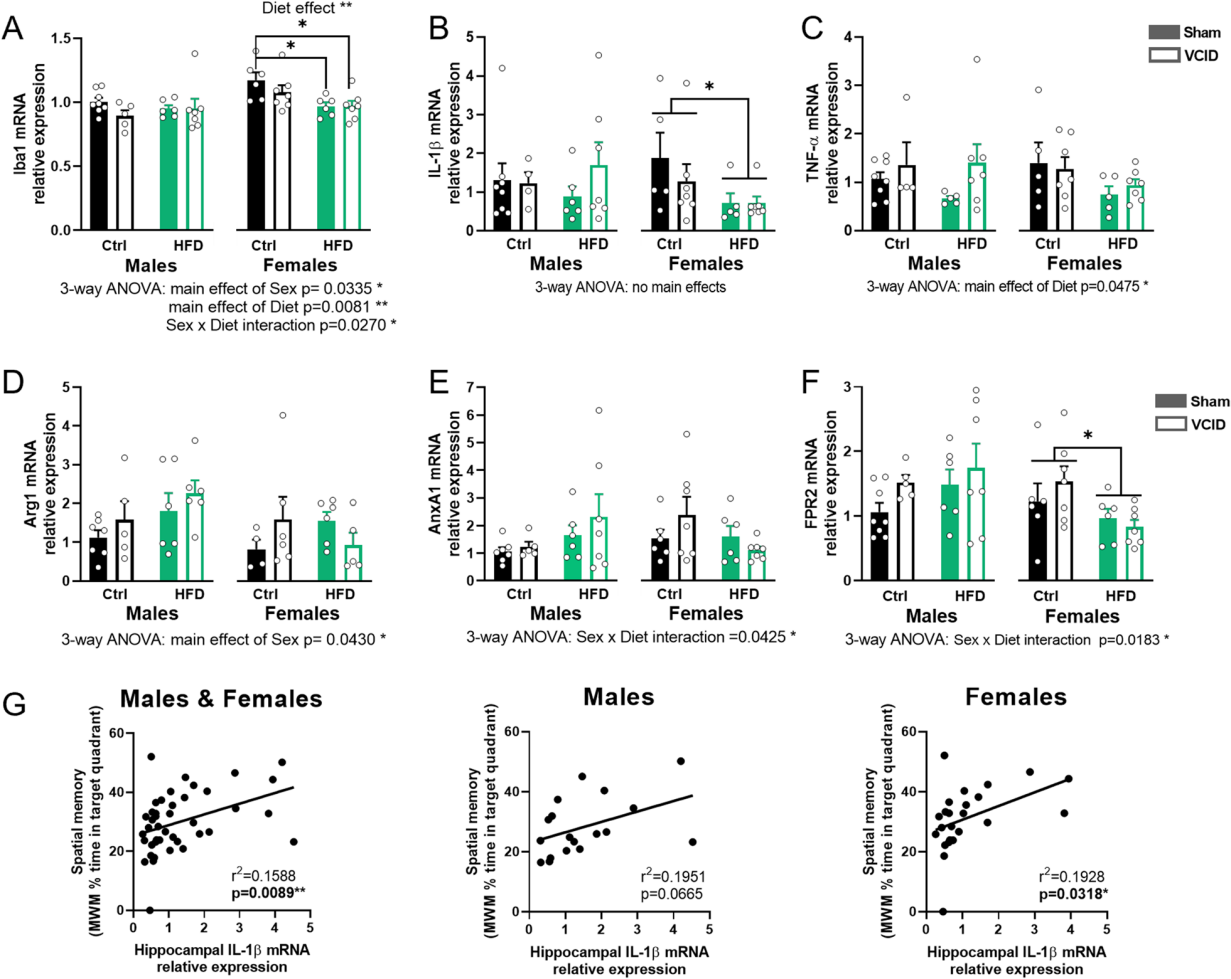
Diet effects on inflammation-related gene changes in the hippocampus are sex-specific. Relative expression of mRNA of Iba1 (**A**), inflammatory cytokines IL-1β (**B**) and TNF-α (**C**), anti-inflammatory factor Arg1 (**D**) and pro-resolution factors: AnxA1 (**E**) and its receptor FPR2 (**F**). mRNA levels were quantified using the ΔΔCq method and reported as fold change relative to the males on a Ctrl diet with a sham surgery. Data are represented as mean + SEM. 2-way ANOVA with Tukey’s post hoc test *p < 0.05 effect of diet. 3-way ANOVA results are reported under the graph *p < 0.05, **p < 0.01. Correlation of IL-1β expression with the % time spent in the target quadrant during the probe trial of the Morris water maze as a readout for spatial memory (**G**). Correlation analyzed using simple linear regression for each sex with *r*^2^ and p values noted for each. *n* = 4–8 mice/group. *Ctrl* control diet, *HFD* high fat diet

Microglia, along with other resident and infiltrating cells, participate in the inflammatory reaction of the CNS to the damage caused by VCID [9]. Chronic neuroinflammation has been linked to cognitive decline in VCID patients, with high expression of IL-1β and TNF-α in the hippocampus [43]. We therefore assessed the expression levels of these pro-inflammatory cytokines as well as Iba1 in the hippocampus via RT-qPCR. In males, we did not detect any significant effect of diet or VCID for Iba1 or either cytokine. However, in females HF diet significantly reduced Iba1 mRNA expression (2-way ANOVA main effect of diet *p* = 0.004). This was supported by a post hoc difference between the Ctrl sham females and HF Sham and VCID females (Tukey’s post hoc *p* = 0.0417 and *p* = 0.0348, respectively). When sexes were directly compared via 3-way ANOVA, a sex × diet interaction was found for Iba1 and (*p* = 0.027) that was driven by a reduction within HF-fed females, but not HF males, relative to their controls (Fig. 4A). Similar diet effects were observed in females for IL-1β mRNA expression (2-way ANOVA main effect of diet *p* = 0.0379, Fig. 4B). No significant differences were observed for the males or females in TNF-α expression, however we found a main effect of diet (3-way ANOVA *p* = 0.0475; Fig. 4C).

#### Anti-inflammatory factors

When activated, microglia and infiltrating macrophages can be pro-inflammatory and secrete pro-inflammatory cytokines or can be in an anti-inflammatory state, characterized by the expression of several markers, such as arginase1 (Arg1) [44–47]. When examining the expression level of Arg1 mRNA, we did not find any effect of diet or VCID on males or females independently. However, we found a main effect of sex  when comparing sexes (3-way ANOVA, *p* = 0.043; Fig. 4D). This is probably due to HF diet affecting Arg1 expression differently between sexes by increasing it in males. We also analyzed levels of several other anti-inflammatory genes (FIZZ1, YM1); however, they were below the level of detection or had extremely low of levels of expression (Ct values > 35).

#### Inflammation resolution factors

Inflammation resolution is necessary to clear damaged cells and promote tissue repair [48]. It is regulated by several factors including Annexin A1 (AnxA1) and its receptor formyl peptide receptor 2 (FPR2). AnxA1–FPR2 signaling has been shown to initiate pro-resolving pathways to bring about a return to tissue homeostasis after vascular injury [49]. For males, we did not detect any significant effect of diet or VCID for both factors. For females, no significant differences were observed for Anxa1 expression. However, HF diet significantly reduced FPR2 mRNA expression (2-way main effect of diet *p* = 0.0279). When sexes were directly compared via 3-way ANOVA, for both AnxA1 and FPR2, there was a sex × diet interaction (*p* = 0.0425 and *p* = 0.0183, respectively). This was driven by HF diet-induced decrease in FPR2 in females, but not males (Fig. 4E, F).

Surprisingly, increased IL-1β expression was positively associated with spatial memory as measured by greater time spent in the target quadrant of the Morris water maze (MWM) in all animals (*r*^2^ = 0.1588, *p* = 0.0089; Fig. 4G). This association was also significant in females (*r*^2^ = 0.1928, *p* = 0.0318). No other associations were found between neuroinflammatory or pro-resolving factors and cognitive performance (Additional file 1: Fig. S5). Taken together, these data show that neuroinflammation was influenced by weight gain in a sex-specific manner, wherein HF diet dampened both proinflammatory and resolution mediators in females but not males.

Studies have demonstrated an important role of endothelial cells and pericytes in VCID pathology. Further, these cells in the brain have been shown to be negatively affected by diabetes, leading to changes in cerebrovascular structure, angiogenesis, capillary rarefaction, and altered blood brain barrier (BBB) permeability [50–55]. To preliminarily probe how the prediabetic state of our mice has affected these cells, we examined differences in expression of vasculature-related genes, i.e., CD31 (endothelial cell marker), PDGFRβ (pericyte marker), Claudin 5 (tight junction protein that is associated with BBB integrity). For all 3 genes, we found an effect of diet in females, whereby females fed a HF diet showed decreased levels of mRNA compared to those on control diet (Additional file 1: Fig. S6). This suggests that HF diet may cause changes in cerebrovascular structure in females but not males.

## Discussion

We previously reported sex differences in the effect of diet-induced obesity with prediabetes on cognitive deficits in a middle-aged mouse model of VCID [30]. Using chronic cerebral hypoperfusion to model VCID and a HF diet to induce obesity with prediabetes, we showed that females are more susceptible to metabolic and cognitive effects of a HF diet [30]. Here, we show that these sex differences extend to the underlying neuropathology, specifically white matter changes and neuroinflammation. White matter was more negatively impacted by cerebral hypoperfusion in males and by HF diet in females. In both sexes we do not observe any correlation between white matter changes and cognitive outcomes. Microglia activation was increased by HF diet in males but decreased in females. In both sexes an increase in activated microglia in the hippocampus, was associated with worse episodic-like memory. Thus, in males, HF diet-induced cognitive impairment appears to be partially driven by increases in activated microglia, which were associated with worse cognitive function. The underlying cause of diet-induced cognitive impairment in females remains elusive. Our preliminary observations hint to changes in endothelial density and reduced BBB integrity; however, further studies are required to investigate this potential mechanism. These results highlight sex differences in the effects of diet-induced obesity on white matter and neuroinflammation. These findings further highlight the need to consider sex as a key biological variable in dementia research, and more focused efforts in females specifically.

Sex differences have been described in white matter organization, volume, and damage in neurodegenerative disease, including dementia, in both humans and rodents [56–61]. Here, we show that these differences extend to the effects of VCID and/or HF diet on white matter loss. We found that VCID impacted white matter loss mainly in males, leading to a decrease of mature oligodendrocytes. This is in line with the literature, describing white matter injury in the CC of young male rodents using several models of VCID [27, 62–65]. Studies investigating the effect of VCID on white matter loss in females are scarce. Our results show metabolically healthy females are generally protected from VCID-induced reductions in white matter in the CC.

When examining the effects of HF diet on white matter, we found a decrease in mature oligodendrocyte density in females but not males in both CC and hippocampus (independent of VCID surgery). Our findings of white matter alterations following high fat diet are in line with both the human and rodent literature. We have previously shown in young male mice that HF diet leads to exacerbated myelin loss and hippocampal atrophy in a VCID model [27, 62]. Obesity, prediabetes and diabetes have all been linked to white matter loss [15, 16, 18, 66]. Obesity is associated with greater white matter hyperintensities in humans in several brain regions, including the CC and hippocampus [15]. Further, obesity leads to accelerated aging of white matter, particularly at mid-life, as middle-aged (~ 50yo) obese individuals showed a decrease in cortical white matter volume similar to the level of older controls (~ 60yo) [18]. Most of these studies, however, do not stratify findings by sex. In addition to obesity, the HF diet used in the current study (with diet onset in mid-life) also induces a prediabetic phenotype (impaired glucose tolerance without overt hyperglycemia) [30, 67]. In humans, prediabetes, specifically small elevations in fasting blood glucose levels and impaired glucose tolerance, has been shown to be associated with microstructural abnormalities in white matter tracts [16]. Insulin resistance, another hallmark of prediabetes, is associated with increased myelin water fraction in cognitively unimpaired adults [68]. Using several transgenic mouse lines, insulin signaling has been shown to be crucial for oligodendrocyte genesis and function [69–73]. In line with these studies, we found a female-specific negative association between glucose intolerance and mature oligodendrocytes labeling density in the CC, supporting that females are more vulnerable to the effects of a HF diet than males. Other studies have hinted at a mechanism, showing that HF diet-induced loss of oligodendrocytes is associated with increases in oxidative stress and mitochondrial dysfunction [74]. Whether similar mechanisms underlie our findings is yet to be investigated, especially in regard to females. Although the diet seemed to have altered the white matter pathology especially in females, we do not find any correlation between this and their behavioral outcome, suggesting that white matter loss or damage may not be the leading underlying cause of cognitive impairment.

Microglia, as resident immune cells of the CNS, play a key role in the neuroinflammatory response to various insults, including cerebrovascular damage. Sex differences have been described in microglia, with female microglia generally having higher phagocytic capacity, greater expression of phagocytosis receptors, and greater expression of cellular repair and inflammatory control genes [75, 76]. Conversely, male microglia in general have higher migration capacity and are more reactive [75, 76]. While we did observe similar patterns of higher phagocytic ameboid microglia in control sham females vs males, these findings did not rise to the level of statistical significance.

HF diet is known to induce microglia activation in various areas of the brain, including the CC and hippocampus in young mice [77–81]. Interestingly, Sherman et al. observed that HF diet induced microglia activation in the CC of male but not female 6-month-old mice, which is in agreement with the sex differences we observed in our 15-month-old mice [77]. We found that HF diet led to a male-specific increase in microglia cell density in the CC and an increase in reactive and phagocytic microglia in both the hippocampus and CC. This sex difference was further exacerbated by a decrease in reactive and phagocytic microglia in the CC of HF-fed females. The effects in males are in line with our previous work in young mice showing that HF diet leads to an increase in microglia (Iba1+% area covered) and phagocytic microglia (Iba1+/CD68+% area covered) in males but not females in the dentate gyrus of the hippocampus [20]. However, the effects we observed of HF diet on female microglia were unexpected given their increased metabolic and cognitive impairment. Microglia and infiltrating macrophages have been known to adopt an anti-inflammatory a state characterized by the expression of several markers including Arg1 [44–47]. We found that Arg1 mRNA expression was increased in males, but decreased in females on a HF diet. This decrease in females may lead to an overall increased proinflammatory status compared to males despite the lower density of ameboid microglia. However, these findings are limited to mRNA changes and need further confirmation. A recent study using a mouse model of cortical photothrombotic ischemic stroke, found more severe ischemic injury exacerbated neuronal damage and an increased the inflammatory response in animals in which Arg1+ microglia/macrophages were depleted [82]. This study used younger animals with no specification of sex. Further investigations are needed to better shed light on the status of microglia, especially since recent studies have shown that the same microglia cell can express both pro- and anti-inflammatory markers at the same time [83].

Less is known about the effects of HF diet on microglia in the context of VCID. We have previously shown in young male mice that a combination of HF diet and VCID led to an increase in glia activation in the CC and hippocampus [27]; however, this was a single-sex study. In the current study, we show that on a HF diet, males show an increase in reactive phagocytic microglia as opposed to females that show a decrease. This is first study assessing sex differences in VCID; however, our findings are in in line with findings in other neurological diseases. In a model of traumatic brain injury, HF diet has also been shown to increase microglia activation in brains of HF-fed male but not female mice [77]. In addition, the microglial response 30 days after traumatic brain injury in HF-fed mice was significantly lower in females compared to males in both the CC and hippocampus [77]. Thus, this data is in line with the similar response we observed 4 months after VCID. With aging, female microglia have been shown to lose their ability to adapt their phagocytic activity to inflammatory conditions in vivo [75]. This might explain why in our middle-aged HF-fed females we did not observe an increase in reactive or phagocytic microglia compared to their controls. Alternatively, the timing of the inflammatory response following VCID could be different in females. Here, we examined outcomes 4 months post-VCID surgery; further longitudinal studies would be needed to investigate sex differences in the timing of the inflammatory response.

Correlations were run to determine relationships between microglia and cognitive function overall and separately by sex. We found a negative association between phagocytic microglia in the hippocampus and episodic-like memory in both sexes. This is supported by a study showing that HF diet-induced cognitive decline was associated with an increase in microglia activation and phagocytic activity (CD68+) in the hippocampus [84]. Synapse phagocytosis by microglia was identified as an underlying mechanism: through pharmacological inhibition of microglial activation, dendritic spine loss and cognitive degradation were prevented [84]. This study was conducted in young male mice, leaving the question of whether a similar mechanism is observed in females unanswered. In this study, however, we show that the response of microglia to HF diet is sex-specific with a surprising decrease in females. Further studies are needed to elucidate the mechanisms by which HF diet affects microglia and how they might differ between sexes.

Similar to the effects of HF diet on microglia, we found sex differences in the effects of HF diet on inflammatory gene expression in the hippocampus. Females fed a HF diet had a decrease in proinflammatory cytokine expression. This finding was surprising, as obesity has been associated with an increase in neuroinflammation [85–89], although the majority of these studies have been conducted exclusively in males or young animals. Studies suggest that having IL-1β levels that are either too high or too low can have detrimental effects on memory. For example, overexpression of IL-1β impairs learning and memory [90, 91]; however, mice lacking IL-1β also have worse spatial memory than control mice [92]. Our data provide further support that very low IL-1β levels may be linked to impaired memory. Indeed, we observed a correlation between decreased levels of IL-1β in the hippocampus and worse MWM performance in female mice. We had previously reported, in these same mice, that females were more susceptible to the metabolic effects of HF diet, as they showed greater visceral fat accumulation [30]. Further, HF diet led to an increased expression of TNF-α mRNA in their visceral fat [30]. This could lead to high levels of circulating TNF-α originating from visceral fat in females which might explain the low levels needed to be produced locally in the brain. However, all these findings were based on RNA expression and may not necessarily be reflected on the protein level. Further experiments are needed to test this hypothesis.

We observed similar sex-specific effects of HF diet on pro-resolving ANXA1/FPR2. ANXA1 was increased in HF diet-fed males compared to females, whereas its receptor, FPR2, was decreased in HF diet females. This might indicate that the HF diet may disrupt the ANXA1–FPR2 signaling pathway differently in females than in males. Much of what is known about the function of ANXA1/FPR2 in the context of metabolic disease is in the periphery. Plasma ANXA1 is increased in patients with type 2 diabetes compared to normoglycemic controls and is positively correlated with degree of metabolic impairment [93]. ANXA1 KO mice fed a HF diet show greater metabolic impairment than HF-fed WT mice [93]. ANXA1 deficiency has been shown to exacerbate renal and kidney pathology and lipid accumulation in diabetic mice, while its mimetic ameliorates these effects [94]. One study showed that insulin-resistant male mice lacking ANXA1 displayed worse vascular dysfunction in peripheral vessels [95]. It is unknown whether similar effects exist in cerebral vessels. One mechanism behind the sex differences we observed might be the influence of sex hormones. ANXA1 is regulated by estrogens in multiple cell types, including microglia. In vitro studies using primary microglia cultures from female mice have shown that estradiol treatment increases production and release of ANXA1, which enhances phagocytic clearance of apoptotic cells by microglia. ANXA1 via its receptor FPR2 has been shown to mitigate cerebrovascular injury by limiting immune cell infiltration and pro-inflammatory thrombotic cytokine production in mouse models of ischemic stroke [96–98]. ANXA1 KO mice exhibit greater inflammation, larger infarct volume and worse cognitive impairment than controls [99]. Further, mice lacking FPR2 have been shown to suffer greater cerebrovascular damage [98]. Similar protective effects of ANXA1 were found in a model of retinopathy, with ANXA1 KO mice having worse outcomes [100]. However, all these studies were conducted exclusively in young male mice. Given these pro-resolving actions of ANXA1 in cerebrovascular disease, its reduction in the hippocampus of our HF-fed females could contribute to the worse vascular outcomes (lower CBF) and hippocampal-dependent cognitive deficits we previously observed. Studies have demonstrated an important role of endothelial cells and pericytes in VCID pathology. Further, diabetes is associated with changes in cerebrovascular structure, capillary rarefaction, angiogenesis, and impaired blood brain barrier permeability [50–55]. In support of this, our preliminary results indicated a decrease in expression of endothelial, pericyte, and BBB genes in HF-fed, prediabetic females. Our results suggest that a dysregulated inflammatory response might be an underlying cause of cognitive impairment in females. Future studies are needed to further investigate the relationship between this dysregulation of inflammatory responses in females and impaired vascular function.

## Perspectives and significance

This is the first study to examine sex differences in the interaction between VCID and metabolic disease on neuropathology. We identified several sex-specific pathological outcomes in white matter and neuroinflammation. These data highlight the need for future studies elucidating the causal mechanisms in the development of this pathology, which are likely to be different between males and females. One limitation of the current study is lower than anticipated samples sizes due to premature death or illness of these 15-month-old mice with multiple comorbidities (high fat diet and VCID model). The underlying mechanism of these sex differences could be related to effects of sex hormones, sex chromosomes or differences in the effects of some risk factors that could be sex specific [101–103]. It is also possible that the timing of neuroinflammation and white matter loss following HF diet or chronic cerebral hypoperfusion/VCID onset could differ in males and females. While this study focused on long-term effects, time course studies would also provide valuable information. Aside from the white matter changes and neuroinflammation assessed in the current study, there may be sex differences in other underlying pathologies that were outside the scope of the current study (e.g., synaptic loss, blood brain barrier damage, and/or peripheral immune responses). Further studies are underway that are needed to fill these gaps in knowledge. In a clinical context, the current study adds to our understanding of sex differences in underlying neuropathology of VCID in the presence of a common risk factor (obesity/prediabetes). This information is crucial for the development of effective, sex-specific therapeutic interventions for VCID.

## Supplementary Information

**Additional file 1: Figure S1.** Representative images of white matter labeling. Representative images of myelin basic protein labeling and CC1 labeling are shown for all experimental groups. Scale bar 200 µm. M = males F = females, Ctrl = control diet, HFD = high fat diet. **Figure S2.** Correlations between metabolic and behavior measures with white matter parameters. Pearson correlation matrices representing the relationships between metabolic, cognitive, and white matter in the CCand the hippocampus of males and females combined as well as each sex separately. GTT AUC: area under the curve from the glucose tolerance test, high GTT AUC indicates greater glucose intolerance; MWM % in target quadrant: % of the time spent in the target quadrant of the probe trial of the MWM test, higher percentage indicates better spatial memory; NORI: novel object recognition index, which is calculated as % time spent with the novel object in the testing trial of the NOR test, higher percentage indicates better episodic-like memory. Pearson *r* values are presented in black, p-values are presented in blue font, *p < 0.05, **p < 0.01 significant correlation; Green: positive correlation, Blue: negative correlation. **Figure S3.** Representative images of microglia labeling. Representative images of Iba1 and CD68 labeling are shown for all experimental groups. Scale bar 200 µm. M = males F = females, Ctrl = control diet, HFD = high fat diet. **Figure S4.** Correlations between metabolic and behavior measures with microglia. Pearson correlation matrices representing the relationships between metabolic, cognitive, and Iba1 area density as well as microglial cells in the CCand the hippocampus of males and females combined as well as each sex separately. Iba1 labeling was quantified in the whole hippocampus and cells were counted in the CA1. GTT AUC: area under the curve from the glucose tolerance test, high GTT AUC indicates greater glucose intolerance; MWM % in target quadrant: % of the time spent in the target quadrant of the probe trial of the MWM test, higher percentage indicates better spatial memory; NORI: novel object recognition index, which is calculated as % time spent with the novel object in the testing trial of the NOR test, higher percentage indicates better episodic-like memory. Pearson *r* values are presented in black, *p*-values are presented in blue font, *p < 0.05, ***p < 0.001 significant correlation; Green: positive correlation, Blue: negative correlation. **Figure S5.** Correlations between metabolic and behavior measures with inflammation-related markers expression in the hippocampus. Pearson correlation matrices representing the relationships between metabolic, cognitive, with Iba1, inflammatory markers, anti-inflammatory marker Arg1 and pro-resolution factors in the hippocampus of males and females combined as well as each sex separately. GTT AUC: area under the curve from the glucose tolerance test, high GTT AUC indicates greater glucose intolerance; MWM % in target quadrant: % of the time spent in the target quadrant of the probe trial of the MWM test, higher percentage indicates better spatial memory; NORI: novel object recognition index, which is calculated as % time spent with the novel object in the testing trial of the NOR test, higher percentage indicates better episodic-like memory. Pearson *r* values are presented in black, *p*-values are presented in blue font, *p < 0.05, **p < 0.01 significant correlation; Green: positive correlation, Blue: negative correlation. **Figure S6.** HF diet leads to a decrease in vasculature associated genes in females only. Relative expression of mRNA of CD31, PDGFRβ and Claudin5. mRNA levels were quantified using the ΔΔCq method and reported as fold change relative to the males on a Ctrl diet with a sham surgery. Data are represented as mean + SEM. 2-way ANOVA with Tukey’s post hoc test *p < 0.05, **p < 0.01 effect of diet. 3-way ANOVA results are reported under the graph *p < 0.05, ***p < 0.001. *n* = 5–8 mice/group. Ctrl = control diet, HFD = high fat diet. **Table S1.** 2-way ANOVA Analysis. Results of 2-way ANOVAs examining main effects of diet and VCID. Significant results are shown in white, values trending toward significance in light gray, and non-significant results in dark gray.

## Data Availability

The datasets during and/or analyzed during the current study are available from the corresponding author on reasonable request.

## Abbreviations

AD: Alzheimer’s disease
Arg1: Arginase1
BBB: Blood brain barrier
CBF: Cerebral blood flow
CA1: Cornu ammonis 1
CC1: CC1 epitope of adenomatous polyposis coli, antibody labeling mature oligodendrocytes
CC: Corpus callosum
CNS: Central nervous system
GTT: Glucose tolerance test
HF: High fat
MWM: Morris water maze
MBP: Myelin basic protein
NORI: Novel object recognition index
UCCAO: Unilateral common carotid artery occlusion
VCID: Vascular contributions to cognitive impairment and dementia

## References

[1] Gorelick PB, Scuteri A, Black SE, Decarli C, Greenberg SM, Iadecola C (2011). Vascular contributions to cognitive impairment and dementia: a statement for healthcare professionals from the American heart association/American stroke association. Stroke.

[2] Whitmer RA, Gunderson EP, Quesenberry CP, Zhou J, Yaffe K (2007). Body mass index in midlife and risk of Alzheimer disease and vascular dementia. Curr Alzheimer Res.

[3] Eringa EC, Serne EH, Meijer RI, Schalkwijk CG, Houben AJ, Stehouwer CD (2013). Endothelial dysfunction in (pre)diabetes: characteristics, causative mechanisms and pathogenic role in type 2 diabetes. Rev Endocr Metab Disord.

[4] McCrimmon RJ, Ryan CM, Frier BM (2012). Diabetes and cognitive dysfunction. Lancet.

[5] Biessels GJ, Staekenborg S, Brunner E, Brayne C, Scheltens P (2006). Risk of dementia in diabetes mellitus: a systematic review. Lancet Neurol.

[6] Brannick B, Dagogo-Jack S (2018). Prediabetes and cardiovascular disease: pathophysiology and interventions for prevention and risk reduction. Endocrinol Metab Clin N Am.

[7] CDC. National diabetes statistics report, 2017. Centers for Disease Control and Preventation; 2017. https://www.cdc.gov/diabetes/basics/diabetes.html. Accessed 06 Jan 2017.

[8] Xue M, Xu W, Ou Y-N, Cao X-P, Tan M-S, Tan L (2019). Diabetes mellitus and risks of cognitive impairment and dementia: a systematic review and meta-analysis of 144 prospective studies. Ageing Res Rev.

[9] Poh L, Sim WL, Jo DG, Dinh QN, Drummond GR, Sobey CG (2022). The role of inflammasomes in vascular cognitive impairment. Mol Neurodegener.

[10] Iadecola C (2013). The pathobiology of vascular dementia. Neuron.

[11] Samara A, Murphy T, Strain J, Rutlin J, Sun P, Neyman O (2019). Neuroinflammation and white matter alterations in obesity assessed by diffusion basis spectrum imaging. Front Hum Neurosci.

[12] Rosenberg GA (2009). Inflammation and white matter damage in vascular cognitive impairment. Stroke.

[13] Dekkers IA, Jansen PR, Lamb HJ (2019). Obesity, brain volume, and white matter microstructure at MRI: a cross-sectional UK biobank study. Radiology.

[14] Haltia LT, Viljanen A, Parkkola R, Kemppainen N, Rinne JO, Nuutila P (2007). Brain white matter expansion in human obesity and the recovering effect of dieting. J Clin Endocrinol Metab.

[15] Han YP, Tang X, Han M, Yang J, Cardoso MA, Zhou J (2021). Relationship between obesity and structural brain abnormality: accumulated evidence from observational studies. Ageing Res Rev.

[16] Jing J, Zhou Y, Pan Y, Cai X, Zhu W, Zhang Z (2022). Reduced white matter microstructural integrity in prediabetes and diabetes: a population-based study. EBioMedicine.

[17] Lampe L, Zhang R, Beyer F, Huhn S, KharabianMasouleh S, Preusser S (2019). Visceral obesity relates to deep white matter hyperintensities via inflammation. Ann Neurol.

[18] Ronan L, Alexander-Bloch AF, Wagstyl K, Farooqi S, Brayne C, Tyler LK (2016). Obesity associated with increased brain age from midlife. Neurobiol Aging.

[19] Robison LS, Gannon OJ, Thomas MA, Salinero AE, Abi-Ghanem C, Poitelon Y (2020). Role of sex and high-fat diet in metabolic and hypothalamic disturbances in the 3xTg-AD mouse model of Alzheimer’s disease. J Neuroinflamm.

[20] Robison LS, Albert NM, Camargo LA, Anderson BM, Salinero AE, Riccio DA (2020). High-fat diet-induced obesity causes sex-specific deficits in adult hippocampal neurogenesis in mice. eNeuro.

[21] Gannon OJ, Robison LS, Salinero AE, Abi-Ghanem C, Mansour FM, Kelly RD (2022). High-fat diet exacerbates cognitive decline in mouse models of Alzheimer’s disease and mixed dementia in a sex-dependent manner. J Neuroinflamm.

[22] Srodulski S, Sharma S, Bachstetter AB, Brelsfoard JM, Pascual C, Xie XS (2014). Neuroinflammation and neurologic deficits in diabetes linked to brain accumulation of amylin. Mol Neurodegener.

[23] Purkayastha S, Cai D (2013). Neuroinflammatory basis of metabolic syndrome. Mol Metab.

[24] van Dijk G, van Heijningen S, Reijne AC, Nyakas C, van der Zee EA, Eisel UL (2015). Integrative neurobiology of metabolic diseases, neuroinflammation, and neurodegeneration. Front Neurosci.

[25] Moller M, Fredholm S, Jensen ME, Wortwein G, Larsen JR, Vilsboll T (2022). Proinflammatory biomarkers are associated with prediabetes in patients with schizophrenia. CNS Spectr.

[26] Johnson LA, Zuloaga KL, Kugelman TL, Mader KS, Morre JT, Zuloaga DG (2016). Amelioration of metabolic syndrome-associated cognitive impairments in mice via a reduction in dietary fat content or infusion of non-diabetic plasma. EBioMedicine.

[27] Zuloaga KL, Johnson LA, Roese NE, Marzulla T, Zhang W, Nie X (2016). High fat diet-induced diabetes in mice exacerbates cognitive deficit due to chronic hypoperfusion. J Cereb Blood Flow Metab.

[28] Chatterjee S, Peters SA, Woodward M, Mejia Arango S, Batty GD, Beckett N (2016). Type 2 diabetes as a risk factor for dementia in women compared with men: a pooled analysis of 2.3 million people comprising more than 100,000 cases of dementia. Diabetes Care.

[29] Sundermann EE, Thomas KR, Bangen KJ, Weigand AJ, Eppig JS, Edmonds EC (2021). Prediabetes is associated with brain hypometabolism and cognitive decline in a sex-dependent manner: a longitudinal study of nondemented older adults. Front Neurol.

[30] Salinero AE, Robison LS, Gannon OJ, Riccio D, Mansour F, Abi-Ghanem C (2020). Sex-specific effects of high-fat diet on cognitive impairment in a mouse model of VCID. FASEB J.

[31] Bin JM, Harris SN, Kennedy TE (2016). The oligodendrocyte-specific antibody ‘CC1’ binds quaking 7. J Neurochem.

[32] Lee YB, Nagai A, Kim SU (2002). Cytokines, chemokines, and cytokine receptors in human microglia. J Neurosci Res.

[33] Nikolakopoulou AM, Dutta R, Chen Z, Miller RH, Trapp BD (2013). Activated microglia enhance neurogenesis via trypsinogen secretion. Proc Natl Acad Sci USA.

[34] Yang Y, Zhao X, Zhu Z, Zhang L (2022). Vascular dementia: a microglia’s perspective. Ageing Res Rev.

[35] Wong AM, Patel NV, Patel NK, Wei M, Morgan TE, de Beer MC (2005). Macrosialin increases during normal brain aging are attenuated by caloric restriction. Neurosci Lett.

[36] Hendrickx DAE, van Eden CG, Schuurman KG, Hamann J, Huitinga I (2017). Staining of HLA-DR, Iba1 and CD68 in human microglia reveals partially overlapping expression depending on cellular morphology and pathology. J Neuroimmunol.

[37] Bartsch T, Dohring J, Reuter S, Finke C, Rohr A, Brauer H (2015). Selective neuronal vulnerability of human hippocampal CA1 neurons: lesion evolution, temporal course, and pattern of hippocampal damage in diffusion-weighted MR imaging. J Cereb Blood Flow Metab.

[38] Bartsch T, Schonfeld R, Muller FJ, Alfke K, Leplow B, Aldenhoff J (2010). Focal lesions of human hippocampal CA1 neurons in transient global amnesia impair place memory. Science.

[39] Pulsinelli WA, Brierley JB, Plum F (1982). Temporal profile of neuronal damage in a model of transient forebrain ischemia. Ann Neurol.

[40] Lana D, Ugolini F, Giovannini MG (2020). An overview on the differential interplay among neurons-astrocytes-microglia in CA1 and CA3 hippocampus in hypoxia/ischemia. Front Cell Neurosci.

[41] Asgeirsdottir HN, Cohen SJ, Stackman RW (2020). Object and place information processing by CA1 hippocampal neurons of C57BL/6J mice. J Neurophysiol.

[42] Cinalli DA, Cohen SJ, Guthrie K, Stackman RW (2020). Object recognition memory: distinct yet complementary roles of the mouse CA1 and perirhinal cortex. Front Mol Neurosci.

[43] Belkhelfa M, Beder N, Mouhoub D, Amri M, Hayet R, Tighilt N (2018). The involvement of neuroinflammation and necroptosis in the hippocampus during vascular dementia. J Neuroimmunol.

[44] Doyle AG, Herbein G, Montaner LJ, Minty AJ, Caput D, Ferrara P (1994). Interleukin-13 alters the activation state of murine macrophages in vitro: comparison with interleukin-4 and interferon-gamma. Eur J Immunol.

[45] Stein M, Keshav S, Harris N, Gordon S (1992). Interleukin 4 potently enhances murine macrophage mannose receptor activity: a marker of alternative immunologic macrophage activation. J Exp Med.

[46] Muzio L, Viotti A, Martino G (2021). Microglia in neuroinflammation and neurodegeneration: from understanding to therapy. Front Neurosci.

[47] Rossi C, Cusimano M, Zambito M, Finardi A, Capotondo A, Garcia-Manteiga JM (2018). Interleukin 4 modulates microglia homeostasis and attenuates the early slowly progressive phase of amyotrophic lateral sclerosis. Cell Death Dis.

[48] Serhan CN, Chiang N, Van Dyke TE (2008). Resolving inflammation: dual anti-inflammatory and pro-resolution lipid mediators. Nat Rev Immunol.

[49] Perretti M, D'Acquisto F (2009). Annexin A1 and glucocorticoids as effectors of the resolution of inflammation. Nat Rev Immunol.

[50] Li W, Prakash R, Kelly-Cobbs AI, Ogbi S, Kozak A, El-Remessy AB (2010). Adaptive cerebral neovascularization in a model of type 2 diabetes: relevance to focal cerebral ischemia. Diabetes.

[51] Prakash R, Somanath PR, El-Remessy AB, Kelly-Cobbs A, Stern JE, Dore-Duffy P (2012). Enhanced cerebral but not peripheral angiogenesis in the Goto-Kakizaki model of type 2 diabetes involves VEGF and peroxynitrite signaling. Diabetes.

[52] Borowsky IW, Collins RC (1989). Metabolic anatomy of brain: a comparison of regional capillary density, glucose metabolism, and enzyme activities. J Comp Neurol.

[53] Mankovsky BN, Metzger BE, Molitch ME, Biller J (1996). Cerebrovascular disorders in patients with diabetes mellitus. J Diabetes Complicat.

[54] Prakash R, Johnson M, Fagan SC, Ergul A (2013). Cerebral neovascularization and remodeling patterns in two different models of type 2 diabetes. PLoS ONE.

[55] Edgerton-Fulton M, Ergul A (2022). Vascular contributions to cognitive impairment/dementia in diabetes: role of endothelial cells and pericytes. Am J Physiol Cell Physiol.

[56] Abi Ghanem C, Degerny C, Hussain R, Liere P, Pianos A, Tourpin S (2017). Long-lasting masculinizing effects of postnatal androgens on myelin governed by the brain androgen receptor. PLoS Genet.

[57] Alqarni A, Jiang J, Crawford JD, Koch F, Brodaty H, Sachdev P (2021). Sex differences in risk factors for white matter hyperintensities in non-demented older individuals. Neurobiol Aging.

[58] Bielecki B, Mattern C, Ghoumari AM, Javaid S, Smietanka K, Abi Ghanem C (2016). Unexpected central role of the androgen receptor in the spontaneous regeneration of myelin. Proc Natl Acad Sci USA.

[59] Daoust J, Schaffer J, Zeighami Y, Dagher A, Garcia-Garcia I, Michaud A (2021). White matter integrity differences in obesity: a meta-analysis of diffusion tensor imaging studies. Neurosci Biobehav Rev.

[60] Ghoumari AM, Abi Ghanem C, Asbelaoui N, Schumacher M, Hussain R (2020). Roles of progesterone, testosterone and their nuclear receptors in central nervous system myelination and remyelination. Int J Mol Sci.

[61] Sachdev PS, Parslow R, Wen W, Anstey KJ, Easteal S (2009). Sex differences in the causes and consequences of white matter hyperintensities. Neurobiol Aging.

[62] Zuloaga KL, Zhang W, Yeiser LA, Stewart B, Kukino A, Nie X (2015). Neurobehavioral and imaging correlates of hippocampal atrophy in a mouse model of vascular cognitive impairment. Transl Stroke Res.

[63] Yoshizaki K, Adachi K, Kataoka S, Watanabe A, Tabira T, Takahashi K (2008). Chronic cerebral hypoperfusion induced by right unilateral common carotid artery occlusion causes delayed white matter lesions and cognitive impairment in adult mice. Exp Neurol.

[64] Dominguez R, Zitting M, Liu Q, Patel A, Babadjouni R, Hodis DM (2018). Estradiol protects white matter of male C57BL6J mice against experimental chronic cerebral hypoperfusion. J Stroke Cerebrovasc Dis.

[65] Wakita H, Tomimoto H, Akiguchi I, Matsuo A, Lin JX, Ihara M (2002). Axonal damage and demyelination in the white matter after chronic cerebral hypoperfusion in the rat. Brain Res.

[66] Wang DQ, Wang L, Wei MM, Xia XS, Tian XL, Cui XH (2020). Relationship between type 2 diabetes and white matter hyperintensity: a systematic review. Front Endocrinol (Lausanne).

[67] Salinero AE, Anderson BM, Zuloaga KL (2018). Sex differences in the metabolic effects of diet-induced obesity vary by age of onset. Int J Obes (Lond).

[68] O'Grady JP, Dean DC, Yang KL, Canda CM, Hoscheidt SM, Starks EJ (2019). Elevated insulin and insulin resistance are associated with altered myelin in cognitively unimpaired middle-aged adults. Obesity (Silver Spring).

[69] Joseph D'Ercole A, Ye P (2008). Expanding the mind: insulin-like growth factor I and brain development. Endocrinology.

[70] Liu JP, Baker J, Perkins AS, Robertson EJ, Efstratiadis A (1993). Mice carrying null mutations of the genes encoding insulin-like growth factor I (Igf-1) and type 1 IGF receptor (Igf1r). Cell.

[71] Luzi P, Zaka M, Rao HZ, Curtis M, Rafi MA, Wenger DA (2004). Generation of transgenic mice expressing insulin-like growth factor-1 under the control of the myelin basic protein promoter: increased myelination and potential for studies on the effects of increased IGF-1 on experimentally and genetically induced demyelination. Neurochem Res.

[72] Ye P, Carson J, D'Ercole AJ (1995). In vivo actions of insulin-like growth factor-I (IGF-I) on brain myelination: studies of IGF-I and IGF binding protein-1 (IGFBP-1) transgenic mice. J Neurosci.

[73] Zeger M, Popken G, Zhang J, Xuan S, Lu QR, Schwab MH (2007). Insulin-like growth factor type 1 receptor signaling in the cells of oligodendrocyte lineage is required for normal in vivo oligodendrocyte development and myelination. Glia.

[74] Langley MR, Yoon H, Kim HN, Choi CI, Simon W, Kleppe L (2020). High fat diet consumption results in mitochondrial dysfunction, oxidative stress, and oligodendrocyte loss in the central nervous system. Biochim Biophys Acta Mol Basis Dis.

[75] Yanguas-Casas N, Crespo-Castrillo A, Arevalo MA, Garcia-Segura LM (2020). Aging and sex: impact on microglia phagocytosis. Aging Cell.

[76] Yanguas-Casas N, Crespo-Castrillo A, de Ceballos ML, Chowen JA, Azcoitia I, Arevalo MA (2018). Sex differences in the phagocytic and migratory activity of microglia and their impairment by palmitic acid. Glia.

[77] Sherman M, Liu MM, Birnbaum S, Wolf SE, Minei JP, Gatson JW (2016). Adult obese mice suffer from chronic secondary brain injury after mild TBI. J Neuroinflamm.

[78] Milanova IV, Correa-da-Silva F, Kalsbeek A, Yi CX (2021). Mapping of microglial brain region, sex and age heterogeneity in obesity. Int J Mol Sci.

[79] Erion JR, Wosiski-Kuhn M, Dey A, Hao S, Davis CL, Pollock NK (2014). Obesity elicits interleukin 1-mediated deficits in hippocampal synaptic plasticity. J Neurosci.

[80] Ma YL, Xia JL, Gao X (2018). Suppressing Irf2bp2 expressions accelerates metabolic syndrome-associated brain injury and hepatic dyslipidemia. Biochem Biophys Res Commun.

[81] Thirumangalakudi L, Prakasam A, Zhang R, Bimonte-Nelson H, Sambamurti K, Kindy MS (2008). High cholesterol-induced neuroinflammation and amyloid precursor protein processing correlate with loss of working memory in mice. J Neurochem.

[82] Li T, Zhao J, Gao H (2022). Depletion of Arg1-positive microglia/macrophages exacerbates cerebral ischemic damage by facilitating the inflammatory response. Int J Mol Sci.

[83] Morganti JM, Riparip LK, Rosi S (2016). Call off the dog(ma): M1/m2 polarization is concurrent following traumatic brain injury. PLoS ONE.

[84] Cope EC, LaMarca EA, Monari PK, Olson LB, Martinez S, Zych AD (2018). Microglia play an active role in obesity-associated cognitive decline. J Neurosci.

[85] Van Dyken P, Lacoste B (2018). Impact of metabolic syndrome on neuroinflammation and the blood–brain barrier. Front Neurosci.

[86] Salas-Venegas V, Flores-Torres RP, Rodriguez-Cortes YM, Rodriguez-Retana D, Ramirez-Carreto RJ, Concepcion-Carrillo LE (2022). The obese brain: mechanisms of systemic and local inflammation, and interventions to reverse the cognitive deficit. Front Integr Neurosci.

[87] Zhang X, Dong F, Ren J, Driscoll MJ, Culver B (2005). High dietary fat induces NADPH oxidase-associated oxidative stress and inflammation in rat cerebral cortex. Exp Neurol.

[88] Ugalde-Muniz P, Fetter-Pruneda I, Navarro L, Garcia E, Chavarria A (2020). Chronic systemic inflammation exacerbates neurotoxicity in a Parkinson’s disease model. Oxid Med Cell Longev.

[89] De Souza CT, Araujo EP, Bordin S, Ashimine R, Zollner RL, Boschero AC (2005). Consumption of a fat-rich diet activates a proinflammatory response and induces insulin resistance in the hypothalamus. Endocrinology.

[90] Matousek SB, Hein AM, Shaftel SS, Olschowka JA, Kyrkanides S, O'Banion MK (2010). Cyclooxygenase-1 mediates prostaglandin E(2) elevation and contextual memory impairment in a model of sustained hippocampal interleukin-1beta expression. J Neurochem.

[91] Moore AH, Wu M, Shaftel SS, Graham KA, O'Banion MK (2009). Sustained expression of interleukin-1beta in mouse hippocampus impairs spatial memory. Neuroscience.

[92] Avital A, Goshen I, Kamsler A, Segal M, Iverfeldt K, Richter-Levin G (2003). Impaired interleukin-1 signaling is associated with deficits in hippocampal memory processes and neural plasticity. Hippocampus.

[93] Purvis GSD, Collino M, Loiola RA, Baragetti A, Chiazza F, Brovelli M (2019). Identification of annexinA1 as an endogenous regulator of RhoA, and its role in the pathophysiology and experimental therapy of type-2 diabetes. Front Immunol.

[94] Wu L, Liu C, Chang DY, Zhan R, Zhao M, Man Lam S (2021). The attenuation of diabetic nephropathy by annexin A1 via regulation of lipid metabolism through the AMPK/PPARalpha/CPT1b pathway. Diabetes.

[95] Jelinic M, Kahlberg N, Leo CH, Ng HH, Rosli S, Deo M (2020). Annexin-A1 deficiency exacerbates pathological remodelling of the mesenteric vasculature in insulin-resistant, but not insulin-deficient, mice. Br J Pharmacol.

[96] Ansari J, Kaur G, Gavins FNE (2018). Therapeutic potential of annexin A1 in ischemia reperfusion injury. Int J Mol Sci.

[97] Smith HK, Gil CD, Oliani SM, Gavins FN (2015). Targeting formyl peptide receptor 2 reduces leukocyte-endothelial interactions in a murine model of stroke. FASEB J.

[98] McArthur S, Loiola RA, Maggioli E, Errede M, Virgintino D, Solito E (2016). The restorative role of annexin A1 at the blood-brain barrier. Fluids Barriers CNS.

[99] Gavins FN, Dalli J, Flower RJ, Granger DN, Perretti M (2007). Activation of the annexin 1 counter-regulatory circuit affords protection in the mouse brain microcirculation. FASEB J.

[100] Hui Q, Zheng F, Qin L, Pei C (2022). Annexin A1 promotes reparative angiogenesis and ameliorates neuronal injury in ischemic retinopathy. Curr Eye Res.

[101] Abi-Ghanem C, Robison LS, Zuloaga KL (2020). Androgens’ effects on cerebrovascular function in health and disease. Biol Sex Differ.

[102] Gannon OJ, Robison LS, Custozzo AJ, Zuloaga KL (2019). Sex differences in risk factors for vascular contributions to cognitive impairment & dementia. Neurochem Int.

[103] Robison LS, Gannon OJ, Salinero AE, Zuloaga KL (2019). Contributions of sex to cerebrovascular function and pathology. Brain Res.

