# Supplementary material for: Sex differences in the effects of high fat diet on underlying neuropathology in a mouse model of VCID
Source: Biol Sex Differ. 2023 May 19;14:31. doi: 10.1186/s13293-023-00513-y (PMC10199629; doi:10.1186/s13293-023-00513-y)
Supplement: Supplementary file 1 — Additional file 1: Figure S1. Representative images of white matter labeling. Representative images of myelin basic protein labeling and CC1 labeling are shown for all experimental groups. Scale bar 200 µm. M = males F = females, Ctrl = control diet, HFD = high fat diet. Figure S2. Correlations between metabolic and behavior measures with white matter parameters. Pearson correlation matrices representing the relationships between metabolic, cognitive, and white matter in the CCand the hippocampus of males and females combined as well as each sex separately. GTT AUC: area under the curve from the glucose tolerance test, high GTT AUC indicates greater glucose intolerance; MWM % in target quadrant: % of the time spent in the target quadrant of the probe trial of the MWM test, higher percentage indicates better spatial memory; NORI: novel object recognition index, which is calculated as % time spent with the novel object in the testing trial of the NOR test, higher percentage indicates better episodic-like memory. Pearson r values are presented in black, p-values are presented in blue font, *p < 0.05, **p < 0.01 significant correlation; Green: positive correlation, Blue: negative correlation. Figure S3. Representative images of microglia labeling. Representative images of Iba1 and CD68 labeling are shown for all experimental groups. Scale bar 200 µm. M = males F = females, Ctrl = control diet, HFD = high fat diet. Figure S4. Correlations between metabolic and behavior measures with microglia. Pearson correlation matrices representing the relationships between metabolic, cognitive, and Iba1 area density as well as microglial cells in the CCand the hippocampus of males and females combined as well as each sex separately. Iba1 labeling was quantified in the whole hippocampus and cells were counted in the CA1. GTT AUC: area under the curve from the glucose tolerance test, high GTT AUC indicates greater glucose intolerance; MWM % in target quadrant: % of the time spent in th [file 13293_2023_513_MOESM1_ESM.pptx]

## Slide 1
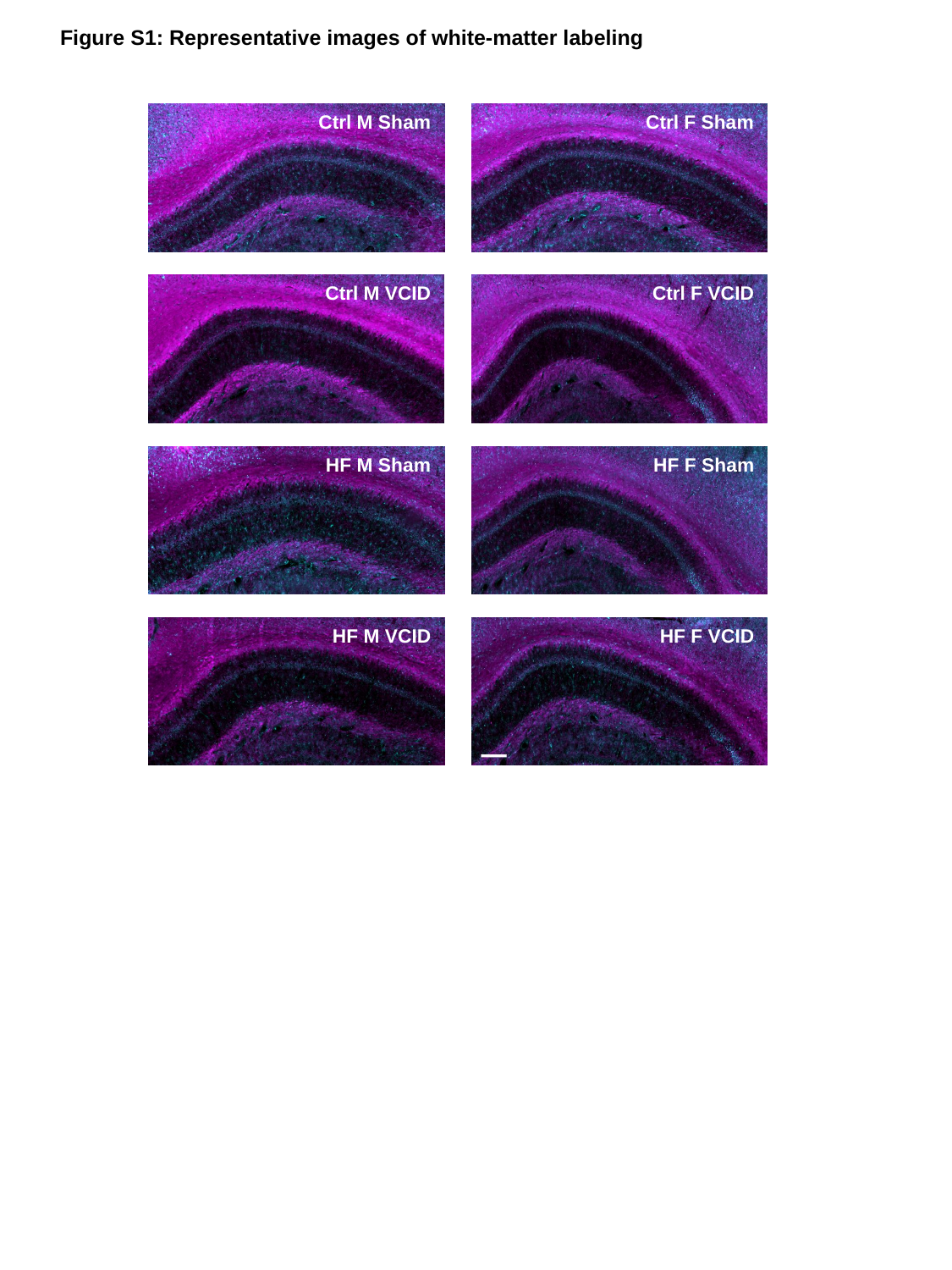

Figure S1: Representative images of white-matter labeling
Ctrl M Sham
Ctrl F Sham
Ctrl M VCID
Ctrl F VCID
HF M Sham
HF F Sham
HF M VCID
HF F VCID

## Slide 2
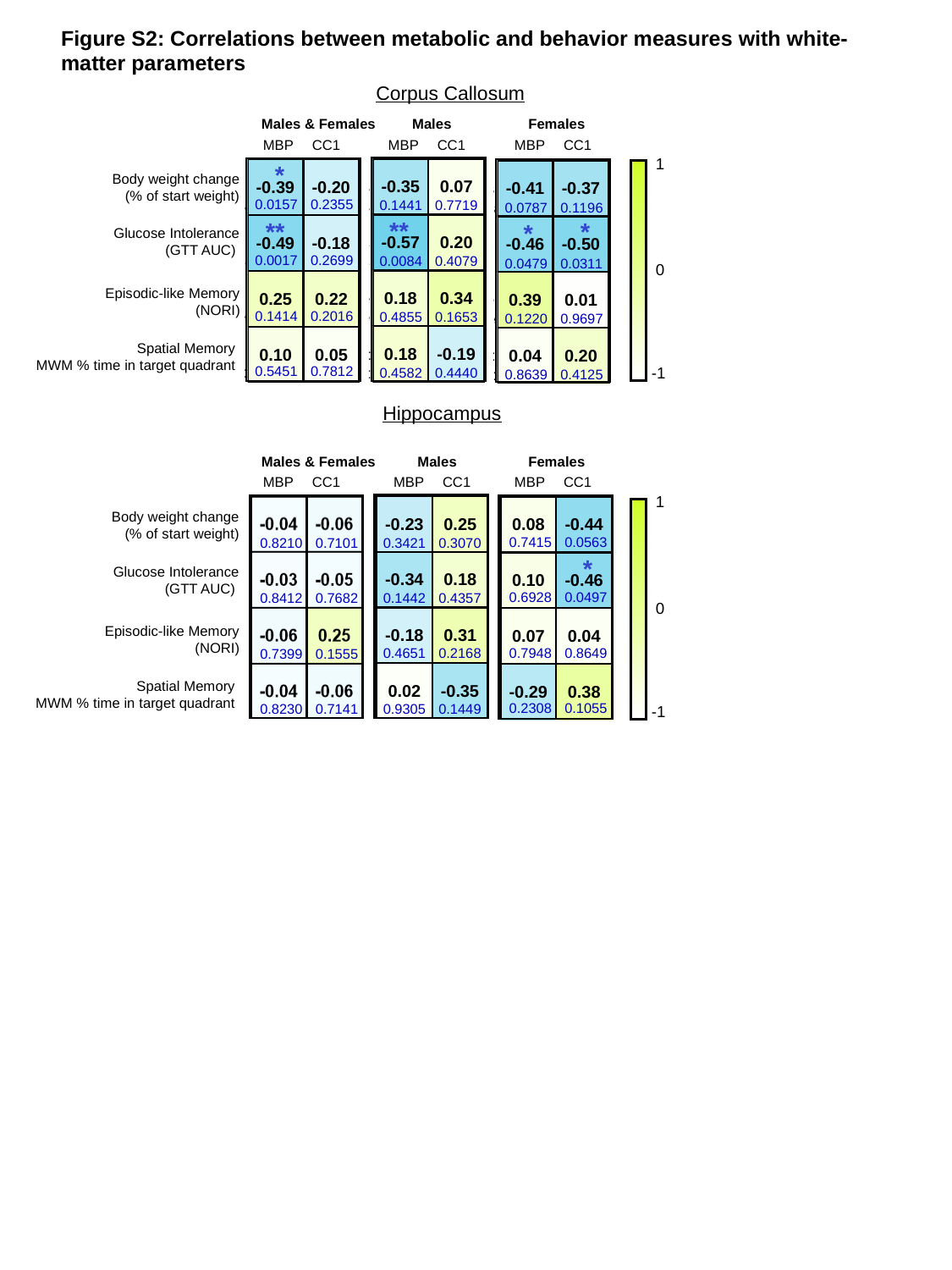

Figure S2: Correlations between metabolic and behavior measures with white-matter parameters
Corpus Callosum
Males & Females
Males
MBP
CC1
**
Females
MBP
CC1
MBP
CC1
 1
 0
-1
*
**
*
*
Body weight change
(% of start weight)
Glucose Intolerance
(GTT AUC)
Episodic-like Memory
 (NORI)
Spatial Memory
MWM % time in target quadrant
Hippocampus
Males & Females
Males
Females
MBP
CC1
MBP
CC1
MBP
CC1
*
 1
 0
-1
Body weight change
(% of start weight)
Glucose Intolerance
(GTT AUC)
Episodic-like Memory
 (NORI)
Spatial Memory
MWM % time in target quadrant

## Slide 3
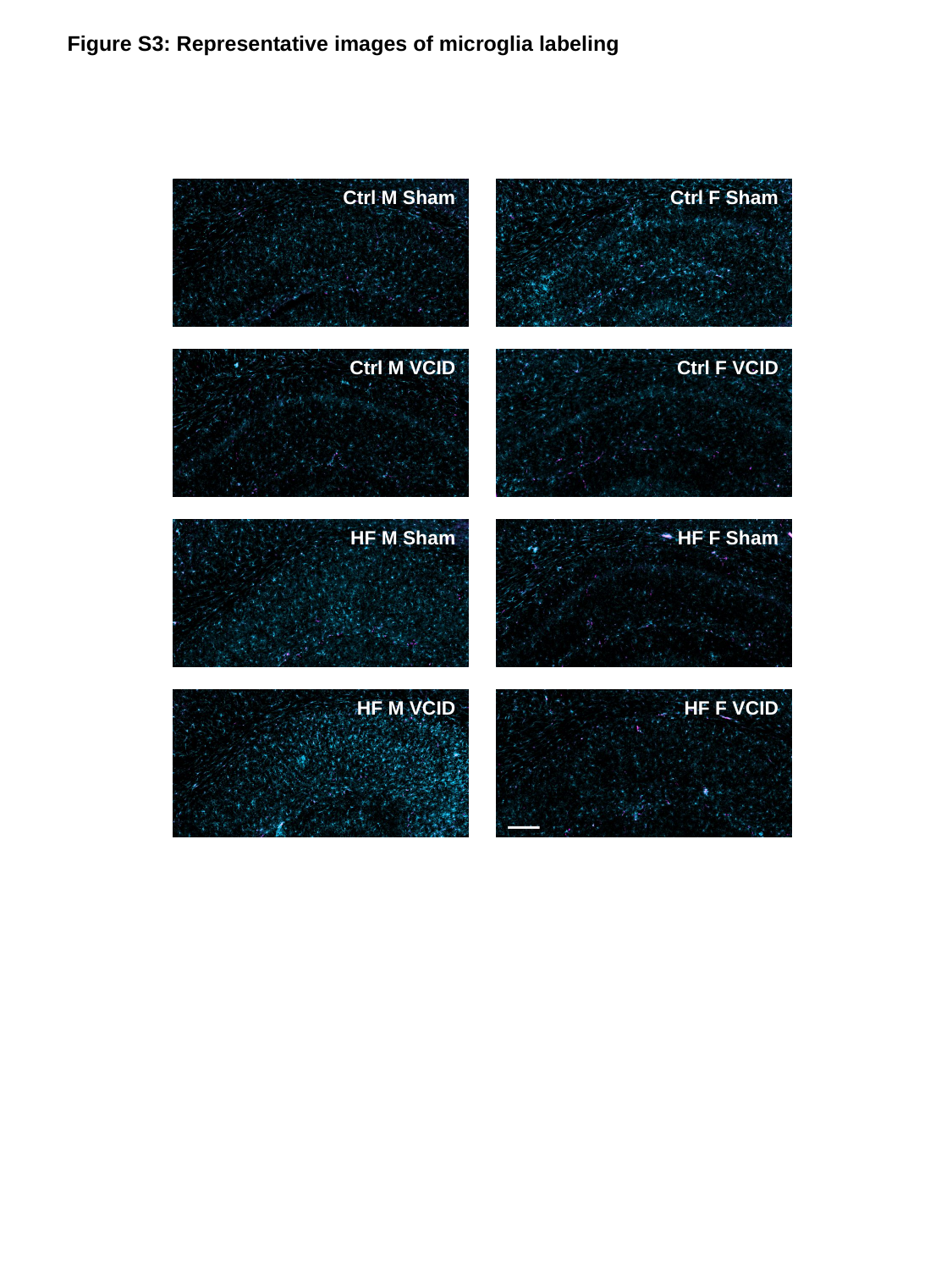

Figure S3: Representative images of microglia labeling
Ctrl M Sham
Ctrl F Sham
Ctrl M VCID
Ctrl F VCID
HF M Sham
HF F Sham
HF M VCID
HF F VCID

## Slide 4
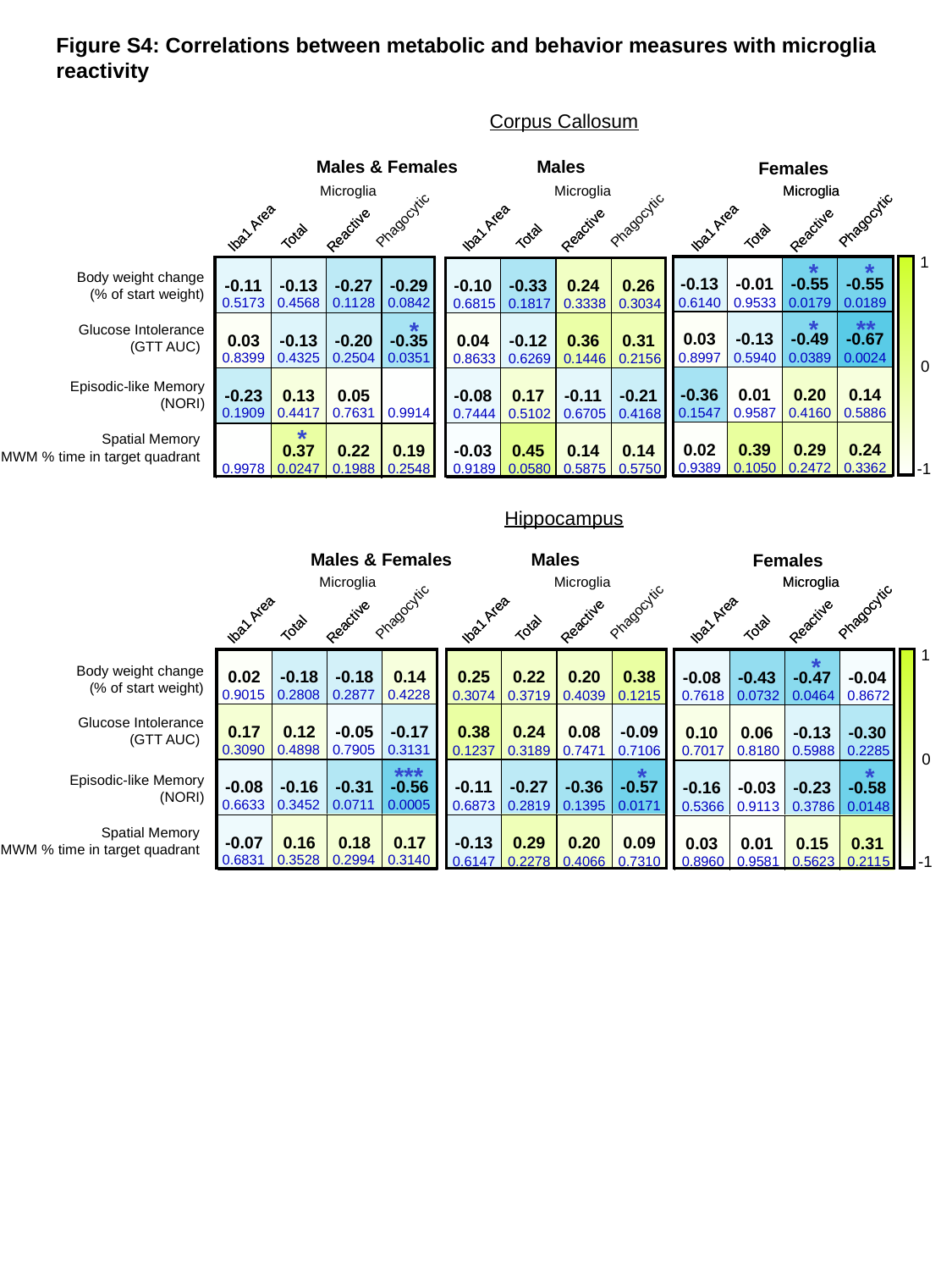

Figure S4: Correlations between metabolic and behavior measures with microglia reactivity
Corpus Callosum
Males & Females
Males
Females
Microglia
Phagocytic
Iba1 Area
Iba1 Area
Reactive
Reactive
Total
Total
Microglia
Phagocytic
Iba1 Area
Iba1 Area
Reactive
Reactive
Total
Total
Microglia
Microglia
Phagocytic
Phagocytic
Iba1 Area
Iba1 Area
Reactive
Reactive
Total
Total
*
*
*
**
 1
 0
-1
*
*
Body weight change
(% of start weight)
Glucose Intolerance
(GTT AUC)
Episodic-like Memory
 (NORI)
Spatial Memory
MWM % time in target quadrant
Hippocampus
Males & Females
Males
Females
Microglia
Phagocytic
Iba1 Area
Iba1 Area
Reactive
Reactive
Total
Total
Microglia
Phagocytic
Iba1 Area
Iba1 Area
Reactive
Reactive
Total
Total
Microglia
Microglia
Phagocytic
Phagocytic
Iba1 Area
Iba1 Area
Reactive
Reactive
Total
Total
 1
 0
-1
*
*
*
***
Body weight change
(% of start weight)
Glucose Intolerance
(GTT AUC)
Episodic-like Memory
 (NORI)
Spatial Memory
MWM % time in target quadrant

## Slide 5
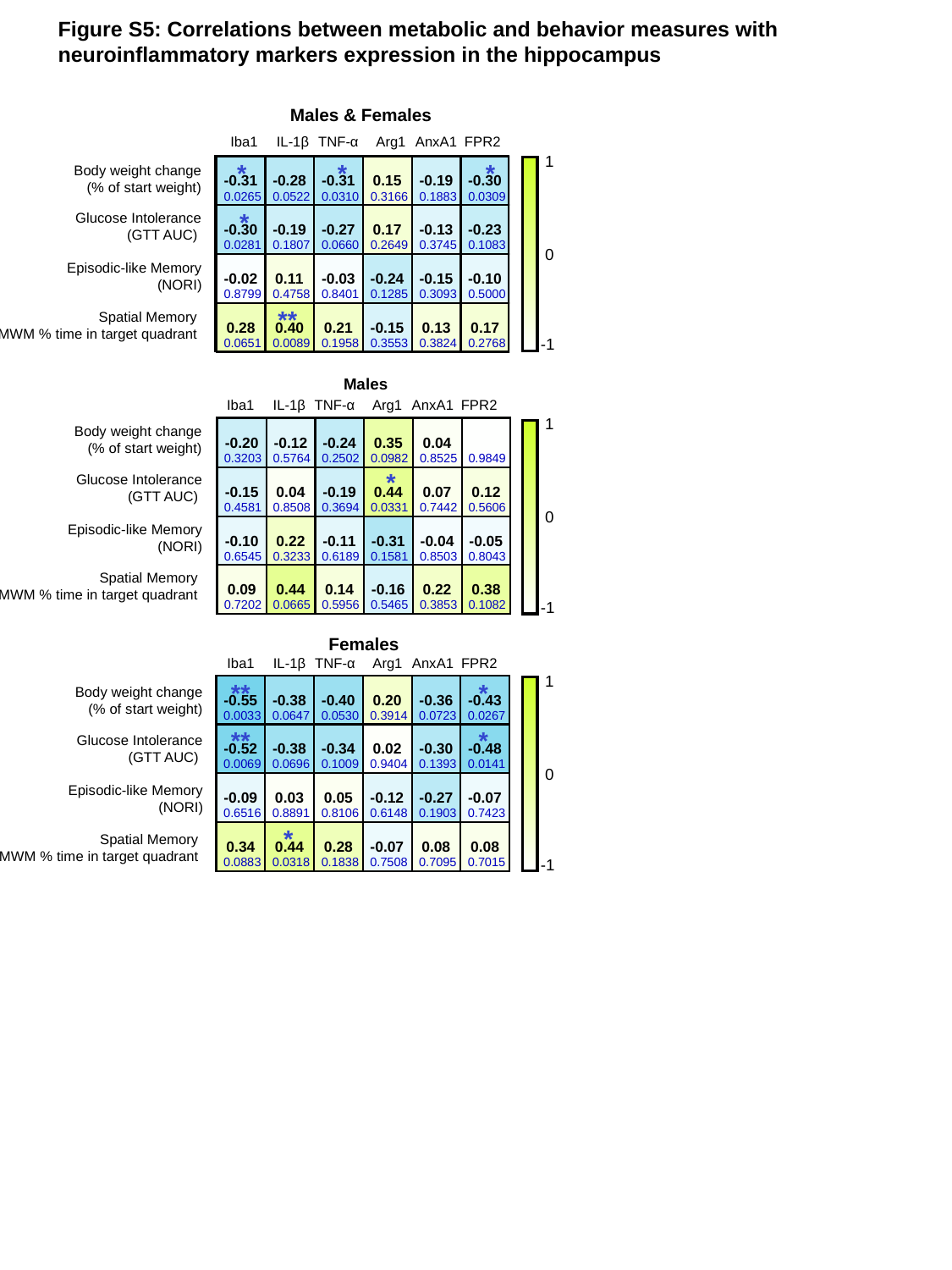

Figure S5: Correlations between metabolic and behavior measures with neuroinflammatory markers expression in the hippocampus
Males & Females
Iba1
IL-1β
TNF-α
Arg1
AnxA1
FPR2
*
*
*
*
**
 1
 0
-1
Body weight change
(% of start weight)
Glucose Intolerance
(GTT AUC)
Episodic-like Memory
 (NORI)
Spatial Memory
MWM % time in target quadrant
Males
Iba1
IL-1β
TNF-α
Arg1
AnxA1
FPR2
*
 1
 0
-1
Body weight change
(% of start weight)
Glucose Intolerance
(GTT AUC)
Episodic-like Memory
 (NORI)
Spatial Memory
MWM % time in target quadrant
Females
Iba1
IL-1β
TNF-α
Arg1
AnxA1
FPR2
*
**
**
*
*
 1
 0
-1
Body weight change
(% of start weight)
Glucose Intolerance
(GTT AUC)
Episodic-like Memory
 (NORI)
Spatial Memory
MWM % time in target quadrant

## Slide 6
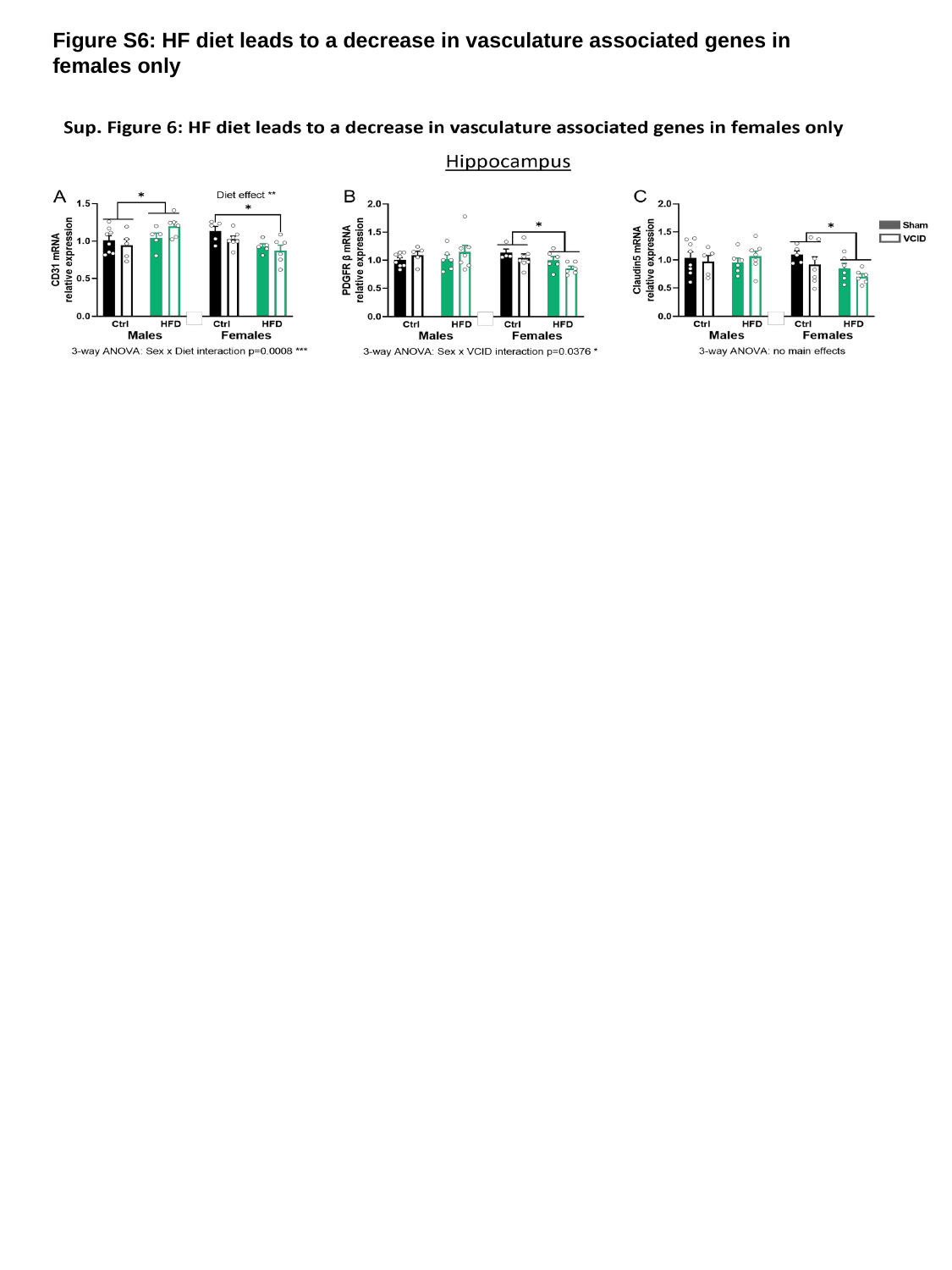

Figure S6: HF diet leads to a decrease in vasculature associated genes in females only

## Slide 7
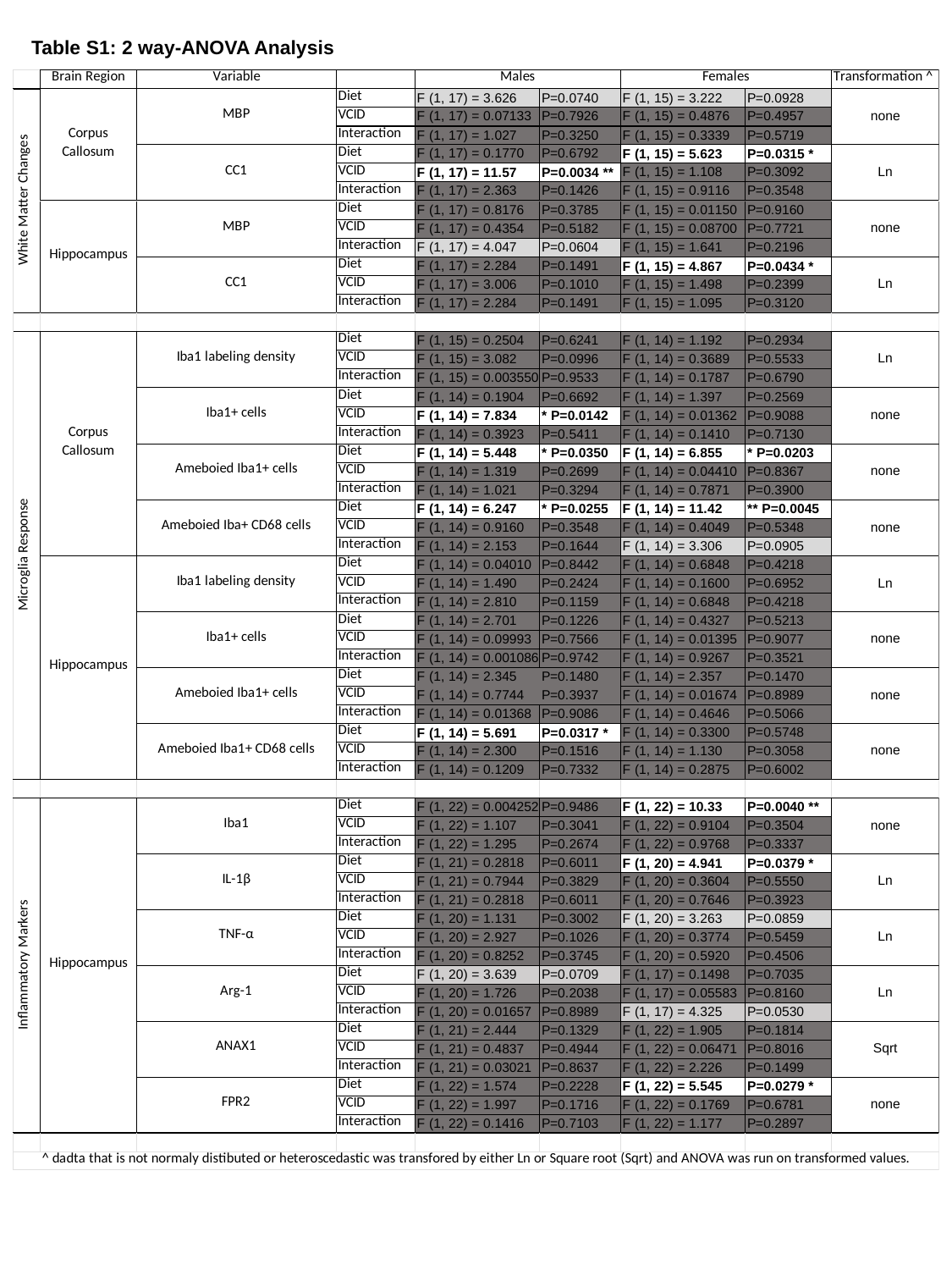

Table S1: 2 way-ANOVA Analysis
